# Role of lncSLCO1C1 in gastric cancer progression and resistance to oxaliplatin therapy

**DOI:** 10.1002/ctm2.691

**Published:** 2022-04-26

**Authors:** Yu‐Feng Xiao, Bo‐Sheng Li, Jing‐Jing Liu, Su‐Min Wang, Jiao Liu, Huan Yang, Yi‐Yang Hu, Chun‐Li Gong, Ji‐Liang Li, Shi‐Ming Yang

**Affiliations:** ^1^ Department of Gastroenterology Xinqiao Hospital Third Military Medical University Chongqing China; ^2^ Wenzhou Medical University Eye Hospital and School of Biomedical Engineering China; ^3^ Cancer Research Centre University of Chinese Academy of Sciences Wenzhou Institute Wenzhou China; ^4^ Institute of Translational and Stratified Medicine University of Plymouth Faculty of Medicine and Dentistry Plymouth UK

## Abstract

**Background:**

Gastric carcinoma (GC) is one of the most deadly diseases due to tumour metastasis and resistance to therapy. Understanding the molecular mechanism of tumour progression and drug resistance will improve therapeutic efficacy and develop novel intervention strategies.

**Methods:**

Differentially expressed long non‐coding RNAs (lncRNAs) in clinical specimens were identified by LncRNA microarrays and validated in different clinical cohorts by quantitative real‐time polymerase chain reaction (qRT‐PCR), in situ hybridisation and bioinformatics analysis. Biological functions of lncRNA were investigated by using cell proliferation assays, migration assays, xenograft tumour models and bioinformatics analysis. Effects of lncSLCO1C1 on GC cell survival were assessed by comet assays and immunofluorescence assays. Underlying molecular mechanisms were further explored by using a number of technologies including RNA pull‐down, mass spectrometry analysis, RNA immunoprecipitation, co‐immunoprecipitation, miRNA sequencing, luciferase reporter assays and molecular modelling.

**Results:**

LncSLCO1C1 was highly upregulated in GC tissue samples and associated with GC patients’ poor overall survival. Overexpression of lncSLCO1C1 promoted proliferation and migration, whereas decreased lncSLCO1C1 expression produced the opposite effects. lncSLCO1C1 also mediated tumour resistance to chemotherapy with oxaliplatin by reducing DNA damage and increasing cell proliferation. Despite sequence overlapping between lncSLCO1C1 and PDE3A, alternations of PDE3A expression had no effect on the GC cell progression, indicating that lncSLCO1C1, not PDE3A, related with the progression of GC cells. Mechanistically, lncSLCO1C1 serves as a scaffold for the structure‐specific recognition protein 1 (SSRP1)/H2A/H2B complex and regulates the function of SSRP1 in reducing DNA damage. Meanwhile, lncSLCO1C1 functions as a sponge to adsorb miR‐204‐5p and miR‐211‐5p that target SSRP1 mRNA, and thus increases SSRP1 expression. Patients with high expressions of both lncSLCO1C1 and SSRP1 have poor overall survival, highlighting the role of lncSLCO1C1 in GC progression.

**Conclusions:**

LncSLCO1C1 promotes GC progression by enhancing cell growth and preventing DNA damage via interacting and scaffolding the SSRP1/H2A/H2b complex and absorbing both miR‐211‐5p and miR‐204‐5p to increase SSRP1 expression.

## Graphical abstract

LncSLCO1C1 promotes tumour progression and resistance to oxaliplatin therapy by enhancing cell growth and preventing DNA damage through interacting and scaffolding the SSRP1/H2A/H2B complex in nucleus and absorbing both miR‐211‐5p and miR‐204‐5p in cytoplasm to increase the expression of SSRP1.

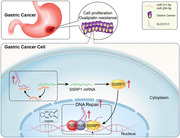

## 1 BACKGROUND

Gastric carcinoma (GC) is the second most common cancer and second leading cause of cancer death in China.^1^, ^2^ The majority of GC patients suffered with advanced stages and are treated by chemotherapy, having a poor overall 5‐year survival. Platinum drugs are commonly used in clinic as the front‐line therapeutic treatment for advanced GC.^1^, ^2^ Platinum‐DNA adducts instigate DNA damage and activate the apoptotic pathway, resulting in cell death.^3^ However, even with platinum‐based combinations, the median overall survival of patients is still poor due to tumour resistance to chemotherapy.^2^, ^4^ Previous studies have suggested many factors including epigenetic alternations and decreased DNA damage are highly associated with tumour resistance to the chemotherapy.^5^, ^6^ The histone chaperone facilitates chromatin transcription (FACT), consists SPT16 and structure‐specific recognition protein 1 (SSRP1), has been identified as a novel target for anticancer therapy.^7^ SSRP1 plays critical roles in reducing DNA damage by interacting with H2A/H2B.^8^, ^9^, ^10^ SSRP1 silencing has been shown to increase sensitivity to platinum and improve γH2AX expression.^11^ The expression of SSRP1 has been reported to be significantly upregulated in several kinds of cancers including breast, lung and pancreatic tumours, and highly associated with patient poor survival.^10^, ^12^ Nevertheless, the function of SSRP1 in GC progression remains largely unknown.

Only 2% of RNA transcripts are believed to be translated into proteins.^13^ Long non‐coding RNAs (lncRNAs) are a class of single‐stranded RNA more than 200 nucleotides, incapable of encoding proteins. lncRNAs were initially considered as transcript noises but later found to play an important role in tumour development.^14^, ^15^ Recently, increasing studies has shown that lncRNAs are able to interact with protein complexes and thus enhance their function in cancer cells. In the nuclear, lncRNAs regulate gene expression by interacting with transcription factors, altering chromatin structure and regulating nuclear body organisation^16^, ^17^; whilst lncRNAs in the cytoplasm are able to serve as competitive endogenous RNAs (ceRNAs) to adsorb miRNAs, abolishing translation inhibition and/or degradation of mRNAs by miRNAs.^18^ However, the biological functions of lncRNAs in GC progression and response to chemotherapy are unclear.

In this study, a novel lncRNA in GC was identified by microarray analysis. We found lncSLCO1C1 is significantly upregulated in GC and closely associated with patient overall survival. We demonstrated lncSLCO1C1 increases tumour cell proliferation and migration, and promotes tumour resistance to chemotherapy with oxaliplatin by decreased DNA damage of GC. We revealed that lncSLCO1C1 in nucleus scaffolds the SSRP1/H2A/H2B complex and decreases DNA damage; whereas lncSLCO1C1 in cytoplasm functions as a sponge to adsorb both miR‐211‐5p and miR‐204‐5p and thus increases SSRP1 expression in GC cells.

## 2 METHODS

### 2.1 Study approval

All experimental procedures in human samples were approved by the Ethical Committee of the Third Military Medical University and carried out in accordance with the Declaration of Helsinki, 1975. All patients were fully informed and their written‐informed consents were obtained. All procedures and welfare monitor in animal study were carried out in accordance with the National Institutes of Health Animal Use Guidelines.

### 2.2 Patient specimens

Forty‐nine pairs of tumour tissue versus normal tissue were collected from GC patients when they were first diagnosed at Xinqiao Hospital during the period from January 2012 to December 2015 (Cohort 1). The diagnosis of GCs was determined by an experienced pathologist (Table S1). Ninety pairs of gastric cancer tissue versus normal tissue in a tissue array (HStm‐Ade180Sur‐07) (Cohort 2) was purchased from Outdo Biotech Co., Ltd. (Shanghai, China) (http://www.superchip.com.cn/). Clinicopathological information in the array and local samples were categorised in accordance with the American Joint Committee on Cancer (AJCC) 2010 TNM classification (Table S2).

### 2.3 Cell lines and culture

GES‐1, SGC7901, BGC823, HGC27, MKN28, MKN74, MKN45 and HEK293 were purchased from and authenticated by the Type Culture Collection of the Chinese Academy of Sciences (Shanghai, China). Cell identification certificate were listed in Supporting Information Material 1. All cells were cultured in Dulbecco's Modified Eagle Medium (Gibco, USA) supplemented with 10% Fetal Bovine Serum (FBS) (Gibco). The cells were transfected with different plasmids or infected with lentivirus for functional assays. SiRNAs against SSRP1 were obtained from GeneChem (Shanghai, China). Short hairpin RNAs against human lncRNAs (lncSLCO1C1) and SSRP1 were purchased from GenePharma (Shanghai, China), were transfected into target GC cells using Lipofectamine 2000, whilst non‐specific shRNA used as a control. In addition, both lncSLCO1C1 and SSRP1‐supression or ‐overexpression lentiviruses were obtained from GeneChem.

### 2.4 Bioinformatics analysis

The whole cell RNA, extracted from BGC823 sh‐lncRNA group and NC group (lnc‐SLCO1C1 knockout cell lines and control cell lines, each group contain three samples), was sent to Novogene Bioinformation Company (Beijing, China) for mRNA expression detection. Gene set enrichment analysis software was applied to analyse genome enrichment in two groups of cell lines. The target genes or signal pathways were chosen by following rules: | normalised enrichment score (NES)| > 1, nominal *p*‐value (NOM *p*‐val) < .05, false discovery rate (FDR) *q*‐val < 0.25. Heatmap drawings are produced using R software platform (https://www.r‐project.org/).

To further explore the target adsorbed miRNA on lncRNA SLCO1C1, full‐length biotin‐labelled lncSLCO1C1 was obtained by in vitro transcription. The full‐length lncRNA was transfected into SGC7901 cells and RNA pull‐down was applied. The streptavidin beads, which contains the target miRNAs, were set to NovelBio Company (Shanghai, China) for miRNA enrichment detection (Data S1).

### 2.5 CCK‐8 assay

An initial density of 3 × 10^3^ (per well) cells were placed into 96‐well plates. Ten microlitres of CCK‐8 (Dojindo, Japan) solution was added into the each well at fixed time points. After 2 h incubation in a cell incubator, detection was carried out.

### 2.6 In situ hybridisation assay

Fluorescence in situ hybridisation (FISH) was carried out using a FISH kit from BersinBio Company (Guangzhou, China). Both green‐ and red‐fluorescent‐labelled probes for lncSLCO1C1 were purchased from BersinBio Company. The location and expression of lncSLCO1C1 in GC tissues and normal tissues were detected by FISH. To detect the location of lncSLCO1C1 in GC cells, FISH was performed on a 20‐mm slide after cells were fixed. FISH was analysed by using a Leica confocal microsystem (Heidelberg, Germany). In addition, a lncRNA in situ hybridisation (ISH) kit (Boster Company, Wuhan, China) was also applied to detect the expression of lncSLCO1C1 in the GC tissue microarray (HStm‐Ade180Sur‐07) (Shanghai Outdo Biotech Co., Ltd.), and analysed by the IXplore Inverted Imaging Platform (Olympus Corporation, Japan).

### 2.7 Immunofluorescence assay

Cells were seeded on cell climbing pieces, followed by fixed by 4% paraformaldehyde, and washed by Phosphate‐Buffered Saline (PBS) solution for three times. Then, cells were incubated with 0.5% Triton X‐100 for 15 min, and blocked in FBS for 60 min. Primary antibodies (γH2AX, purchased from Abcam, Cambridge, MA, USA, ab81299; SSRP1, purchased from Abcam, ab26212; H2A, purchased from Abcam, ab177308; H2B, purchased from Abcam, ab1790) were used and incubated at 4°C overnight. Secondary antibodies (ThermoFisher, MA, USA) were used and incubated at room temperature for 1 h. 4′,6‐diamidino‐2‐phenylindole (DAPI) was used for cell nucleus staining. Pictures were taken by laser confocal microscopy.

### 2.8 Quantitative real‐time PCR

Total RNAs were extracted by TRIzol (cat: 10296010, ThermoFisher), and 1 μg of total RNA was reverse‐transcribed by using the Prime RT Kit. The expression of target genes was detected by quantitative real‐time polymerase chain reaction (qRT‐PCR). The expression of target RNA was normalised to GAPDH. The primers were provided by Sangon Biotech Company (Shanghai, China) (Data S2).

### 2.9 Western blot analysis

Standard Western blot (WB) assays were performed. Anti‐SSRP1 antibody (ab26212), anti‐H2A antibody (ab28155) and anti‐H2B antibody (ab1790) were from Abcam, and anti‐GAPDH antibody was purchased from CST (51332) (Danvers, MA, USA), which was used as a control for whole cell lysates.

### 2.10 In vitro transcription

Sense, antisense, and different truncated segments of lncSLCO1C1 were cloned into pUC57 Plasmids downstream of the T7 promoter. Plasmids were linearised by digestion with *BamHI* (cat: 1010A, Takara, Kusatsu, Shiga, Japan). A T7 in vitro transcription system was applied by using the MAXIscript™ T7 Transcription Kit (cat: AM1312, Thermo Scientific, Waltham, MA, USA), with 1 μg of linearised plasmid as the template. The biotinylated RNA was purified by a MEGAclear™ Transcription Clean‐Up Kit (cat: AM1908, Thermo Scientific).

### 2.11 Transfection of miRNA mimics or inhibitors

Both miRNA mimics (50 nM) and miRNA inhibitors (200 nM) were obtained from GenePharma and used for transfection. RNA oligos were transfected by using Lipofectamine RNAiMAX (cat: 13778075, Invitrogen, CA, USA).

Antisense oligonucleotide (ASO) was purchased from Ribobio (Guangzhou, China). Note that 1X riboFECT™ CP Buffer (v2), ASO storage solution and riboFECT™ CP Reagent (v4) were used for ASO transfection.

### 2.12 Nucleocytoplasmic fractionation assay

PARIS kit (cat: AM1921, Thermo Scientific) was used for this extraction. Cells were collected and re‐suspended by ice‐cold cell fractionation buffer (CFB) for 5–10 min. After centrifugation at 4°C in 500 *g* for 5 min, the supernatant was collected as the cytoplasmic fraction. The nuclear pellet was washed in ice‐cold CFB and fractured by cell disruption buffer for the nuclear sample.

### 2.13 Peritoneal metastasis models

An equal amount (5 × 10^6^) of target cells (SGC7901 NC/shRNA, BGC823 NC/shRNA, MKN28 NC/lncSLCO1C1 or SGC7901DDP NC/shRNA) were injected into the peritoneal cavity of male BALB/c nude mice (Beijing Laboratory Animal Research Centre). After 6 weeks maintained in the Specific Pathogen Free (SPF) condition, all mice were humanely sacrificed. Intestinal tissues collected from the mice were fully unfolded and tumour numbers were counted.

### 2.14 IC50 assay

Cells (4 × 10^5^) were added to each well. Set concentrations of oxaliplatin (0, 1, 2, 4, 8, 16, 32 and 64 μg/ml) were added into the cells and co‐cultured for 48 h. Ten microlitres of CCK‐8 solution was added into each well. The absorbance at 490 nm was measured for each well. The IC50 was calculated using GraphPad Prism 7 (GraphPad Software, Inc., CA, USA).

### 2.15 RNA—RNA immunoprecipitation

The biotin‐labelled lncSLCO1C1 probe was transfected into cells (5 × 10^6^). These cells were cross‐linked with 1% paraformaldehyde for 10 min, then cells were washed, collected and re‐suspended with the lysis buffer (50 Mm Tris–HCl pH 7.0, 10 mM Ethylene Diamine Tetraacetic Acid, 1% Sodium Dodecyl Sulfate (SDS) supplemented with 200 U/ml of a RNAse inhibitor solution, and a cocktail of proteases inhibitor 5 μl/ml). Cell lysates were sonicated and then the nuclear and cytoplasmic RNA were extracted according to PARIS kit (cat: AM1921, Thermo Scientific) instruction. The lysis was combined to magnetic streptavidin beads by co‐incubation at room temperature for 1.5 h, and then the nuclear RNA (10 μg) and the cytoplasmic RNA (10 μg) were, respectively, added and incubated overnight at 4°C. One microgram nuclear RNA and 1 μg cytoplasmic RNA were collected and labelled as “input”. Magnetic streptavidin beads were washed six times. Beads and the “input” samples were incubated with 117 μl immunoprecipitation buffer, 15 μl 10% SDS and 18 μl proteinase K at 55°C for 0.5 h to fully digest the protein. Precipitated RNA was purified, and the miR‐204‐5p and miR‐204‐5p binding to lncSLCO1C1 were detected by qRT‐PCR.

### 2.16 Transwell assays

Target cells were subjected to starvation for 12 h to remove serum influence before performing experiments. Re‐suspended cells were added to the upper chamber of the transwell chamber, whilst medium containing 10% FBS was added to the lower chamber of the 24 well culture plate. The cells in the upper layer of the microporous membrane of the chambers were rinsed two times with PBS, wiped away with a cotton swab. The cells were fixed with 4% paraformaldehyde, and stained with crystal violet solution for 15 min. Pictures were taken under an inverted microscope (Olympus Corporation).

### 2.17 RNA oligoribonucleotides and plasmids

The miRNA duplexes corresponding to mature miR‐204‐5p and miR‐211‐5p were designed as described previously.^19^ The mimics and inhibitors of two miRNAs were obtained from GenePharma.

ENCORI (https://starbase.sysu.edu.cn/) and TargetScan (https://www.targetscan.org/vert_80/) were used to predict both lncRNA SLCO1C1 and SSRP1 3′UTR including one conservative miR‐204‐5p/miR‐211‐5p binding site. A 407bp and 442bp RNA fragment of human lncRNA SLCO1C1 and SSRP1 3′UTR (including miR‐204/211 binding site) was cloned into psiCHECK‐2 vector (Promega, WI, USA). The mutant lncRNA and SSRP1 3′UTR contained the mutated sequence in the binding site of miR‐204/211‐5p. All the fragments were chemically synthesised from Sangon Biotech Company.

PDE3A ORF and lncSCLO1C1 full‐length sequences flanking with flag tag sequence were synthesised (Sangon Biotech Company) and inserted into a pcDNA3.1 vector.

### 2.18 Dual luciferase reporter assays

293T cells were plated and transfected with the indicated wild‐type or mutant reporter plasmids and 50 nM of either miR‐204/miR‐211 or control RNA. After 48 h, cells were collected and analysed using the Dual‐Luciferase Reporter Assay System (Promega). Renilla luciferase activity of each sample was normalised by firefly luciferase activity.

### 2.19 RNA immunoprecipitation

RNA immunoprecipitation (RIP) was performed by using the Magna RIP RNA‐binding Protein Immunoprecipitation Kit (cat: 17‐700, Merck Millipore, MA, USA). SSRP1 antibody (ab26212), anti‐H2A antibody (ab28155) and anti‐H2B antibody (ab1790) for RIP were purchased from Abcam. RNA was detected by qRT‐PCR. The primers used for detecting lncSLCO1C1 are listed in Data S2.

### 2.20 Co‐immunoprecipitation

Co‐immunoprecipitation (Co‐IP) experiments were performed using the Universal Magnetic Co‐IP Kit (cat: 54002, Active Motif, CA, USA) according to the instructions. Anti‐SSRP1 antibody, anti‐H2A antibody and anti‐H2B antibody for IP were purchased from Abcam, and normal mouse IgG (cat: 5946) and rabbit IgG (cat: 6990) were purchased from CST. A WB assay was then performed to detect the interaction of these proteins.

### 2.21 RNA pull‐down assays

According to the instruction,^20^ the whole length of biotinylated lncSLCO1C1 was synthesised in vitro, and purified lncSLCO1C1 was transfected into cells. Cross‐linking with 1% paraformaldehyde for 10 min, cells were washed and collected and re‐suspended with the lysis buffer. Immediately after sonication, lysed samples were centrifuged at 12 000 *g* for 5 min at 4°C. Magnetic streptavidin beads were supplemented and incubated overnight. Then, the beads were collected by a magnetic frame and washed five times. In the isolation step, beads were collected and sent for mass spectrometric detection to explore the possible binding of proteins. Target proteins were also detected by WB.

### 2.22 5′‐RACE and 3′‐RACE assays

A template was synthesised according to the manufacturer's instructions of the GeneRacer™ Kit (cat: L150001, Invitrogen). The 5′‐RACE and 3′‐RACE assays were carried out according to standard procedures. The gene‐specific primers used for the 5′‐RACE and the 3′‐RACE assays are listed in Data S2. The products were electrophoretically analysed with a 1.2% agarose gel, and further sequencing analysis was also performed.

### 2.23 Copy number measurement

Procedure of copy number measurement was described as previous study.^21^ Genomic DNA (gDNA) in GC cells was extracted using an Omega Tissue DNA Kit (Omega (D3396), USA), and gDNA concentration was measured and diluted into six concentrations with 10 folds, which served as the standard templates and used for qPCR to generate a standard curve. The copy number of gDNA was calculated by the formula: copy number = (6.02 × 10^23^ copies/mol) × (ng/μl × 10^–9^)/(2.91 × 10^9^ × 660).

SGC7901 and BGC823 cells were counted about 3 ×  0^6^, and their total RNAs were extracted using a TRIzo (cat: 10296010, ThermoFisher), finally dissolved in 50 μl RNase‐free H_2_O, respectively. The total RNAs were used to amplify lncSLCO1C1, miR‐211‐5p and miR‐204‐5p using qRT‐PCR experiments, and their copy number was calculated according to the standard curve.

### 2.24 Comet assays

Approximately 1 × 10^5^ number of GC cells were collected to perform the comet assays, respectively. Preparation of the bottom and top gel as previous study reported.^22^ The cells were mixed with 50 ml of a lysate buffer (Sangon Biotech Company), which includes 1% Triton X‐100, and incubated overnight at 4°C. The lysates were subject to electrophoresis for 20 min, and then 20 μl Propidium Iodide (PI) solution was added dropwise into each upper gel plate and the plates were placed in a dark condition for 10 min, followed by immersion in 1× PBS for 5 min. The gels were photographed by confocal microscope (Leica (LAS‐AF‐Lite)) and the results were analysed by a Cometscore software (TriTek Corporation, DE, USA).

### 2.25 Tumour xenografts

To detect the role of lncSLCO1C1 in vivo, 4‐week‐old male BALB/c nude mice were obtained from the Beijing Laboratory Animal Research Centre and used in our study. Treated GC cells (5 × 10^6^ cells) were inoculated into the right flank of mice subcutaneously, whilst the same number of control GC cells were injected into the left side of the same mice. Four weeks later, tumours were harvested and paraffin embedded. To explore the function of lncSLCO1C1 in promoting DNA repair of GC cells, oxaliplatin was added to create a DNA damage model in another batch of nude mice. The same amount of target cells and control cells (5 × 10^6^ cells) were injected into the right and left axillae of nude mice to establish the GC xenograft model, respectively. Ten days after subcutaneous injection, oxaliplatin (5 mg/kg) was injected through the abdominal cavity of nude mice every 3 days. The tumours were harvested after 3 weeks.

### 2.26 Statistical analysis

Data were analysed by using Prism 6 (GraphPad Software, Inc.) or SPSS for Windows 16.0.1 software (SPSS Inc., Chicago, IL, USA). All of the data were collected from at least three independent experiments and are presented as means ± standard error (SE). The Shapiro–Wilk test was applied to detect whether the data were normally distributed. If the data from two groups were normally distributed, paired‐sample *t*‐test or independent‐sample *t*‐test was applied to compare the significance, whilst one‐way analysis of variance (ANOVA) was used to compare three or more groups. Overall survival was evaluated by the log‐rank test. Statistical significance was indicated by an asterisk (*p < .05, **p < .01, ***p < .001 and ****p < .0001).

## 3 RESULTS

### 3.1 LncSLCO1C1 is a novel lncRNA upregulated in GC and associated with patient overall survival

**FIGURE 1:**
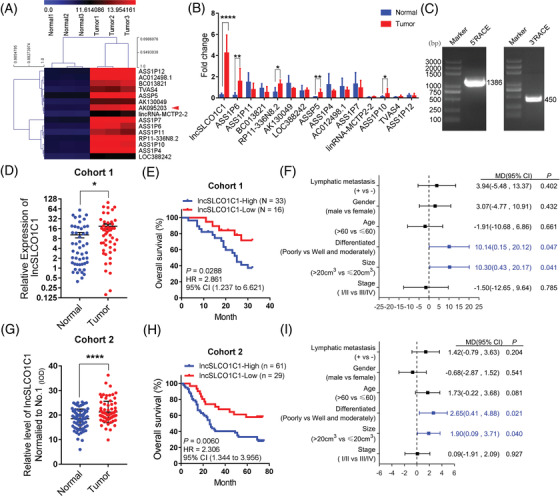
lncSLCO1C1 is a novel long non‐coding RNA (lncRNA) upregulated in gastric carcinoma (GC) and associated with overall survival of GC patients. (A) A heat map generated from the lncRNA microarray showing the top 15 lncRNAs upregulated in three pairs of tumour tissues and adjacent non‐tumour tissues. lncSLCO1C1 is highlighted with red arrow head. (B) Quantitative real‐time polymerase chain reaction (qRT‐PCR) validated the expression of 15 lncRNAs in 20 pairs of GC tissues versus adjacent non‐tumour tissues. β‐Actin served as the internal reference. The data are presented as mean ± standard error of the mean (SEM). The asterisks represented the statistical *p*‐value (*p < .05; **p < .01; ***p < .001; ****p < .0001; Student's test). (C) 5′‐RACE and 3′‐RACE experiments were employed to amplify the full‐length of lncSLCO1C1 (1776 nt) from GC cells. (D) Scatter plots showing the expression of lncSLCO1C1 in Cohort 1 (*n* = 49) was detected by qRT‐PCR with β‐actin as the internal control. The data are presented as mean ± SEM. The asterisks represented the statistical *p*‐value (*p < .05; **p < .01; ***p < .001; ****p < .0001; Student's test). (E) Overall survival analysis showing overall survival of GC patients with high or low expression of lncSLCO1C1 in Cohort 1 (*n* = 49). High expression: the ratio of lncSLCO1C1 expression in tumour tissues and in adjacent non‐tumour tissues in individual pair is >1.0; low expression: the ratio <1.0 (*p* = .0288; log‐rank test). (F) Multivariable clinical analysis showing the correlation between clinical characteristics and lncSLCO1C1 expression in Cohort 1 (*n* = 49). Significant elements are highlighted in blue (the *p*‐value are presented at each group in the figure, Student's test). (G) Scatter plots showing the expression of lncSLCO1C1 in Cohort 2 (*n* = 90) detected by in situ hybridization (ISH) experiments. The data are presented as mean ± SEM. The asterisks represented the statistical *p*‐value (*p < .05; **p < .01; ***p < .001; ****p < .0001; Student's test). (H) Overall survival analysis showing overall survival of GC patients with high or low expression of lncSLCO1C1 in Cohort 2 (*n* = 90). The definition of high/low lncSLCO1C1 expression is described in (E) (*p* = .0288; log‐rank test). (I) Multivariable clinical analysis showing the correlation between clinical characteristics and lncSLCO1C1 expression in Cohort 2 (*n* = 90). Significant elements are highlighted in blue (the *p*‐value are presented at each group in the figure, Student's test)

To identify candidate lncRNAs that have important functions in GC, we initially screened the differential expression of lncRNAs between tumour tissue and adjacent non‐tumour tissue of three pairs of clinical specimens using the Arraystar Human lncRNA Microarray. The resultant lncRNAs were analysed using the Gene Expression Omnibus (GEO) website (GSE58828). Based on stringent criteria (fold change > 2, *p *< .05), we obtained 15 candidate lncRNAs that are significantly upregulated in GC tumour (Figure 1A). We validated this result with 20 pairs of GC tissue versus adjacent non‐tumour tissue by using qRT‐PCR. The expression of ASS1P6, RP11‐336N8.2, ASSP5, ASS1P10 and lncSLCO1C1 was higher in GC tissues, and lncSLCO1C1 was the highest one (Figure 1B). In addition, lncSLCO1C1 also highly expressed in TANRIC database (https://www.tanric.org/) (Figure S1A). We noticed lncSLCO1C1 sequence overlaps the sequence from 6200 nt to 7975 nt within 3′‐UTR of PDE3A mRNA (Figure S1B). We thus designed a pair of primers that specifically bind PDE3A mRNA only (Data S3). qRT‐PCR experiments demonstrated that only lncSLCO1C1 expression but not PDE3A expression is significantly upregulated in Cohort 1 (*n* = 49) (Figures 1D and S1C). We also found that no significant difference was found in PDE3A expression in Cohort 2 (Figure S1D).

We performed 5′‐RACE and 3′‐RACE assays and sequenced the full‐length of lncSLCO1C1 from GC cells (Figures 1C and S1E). LncRNA database (https://lncipedia.org/; http://www.noncode.org/) and ORF Finder (https://www.ncbi.nlm.nih.gov/orffinder/) analysis suggested that lncSLCO1C1 do not encode any protein (Tables S3–S5). To validate this, we made a pair of expression vectors (pcDNA3.1‐lncSLCO1C1‐flag versus pcDNA3.1‐PDE3A‐flag) in which the sequence of flag was conjugated at the 3′‐end of either lncSLCO1C1 or PDE3A. WB experiments showed that flag signal was only detected in BGC823 cells transfected with pcDNA3.1‐PDE3A‐flag but not with pcDNA3.1‐lncSLCO1C1‐flag (Figure S1F), confirming that lncSLCO1C1 does not encode any protein in GC cells.

We then investigated the correlation between lncSLCO1C1 expression and clinical features of GC patients. Overall survival analysis exhibited that GC patients with high level of lncSLCO1C1 have poor overall survival (Figure 1E) whilst PDE3A expression is not associated with GC patient survival (http://bioinfo.henu.edu.cn/GCTCGA). In addition, lncSLCO1C1 expression also correlated with tumour cell poor differentiation and tumour size (Figure 1F; Table S1). LncSLCO1C1 expression signature had an area under the curve (AUC) of 0.6535 for distinguishing GC tissue from matched normal tissue (Figure S1G). As expected, these correlations were also observed in Cohort 2, which contains 90 pairs of GC tissue and non‐tumour tissue (Figures 1G–I and S1H; Table S2) as revealed by ISH experiments, and an AUC of 0.6557 was also obtained (Figure S1I). Altogether, we concluded that lncSLCO1C1 is a novel lncRNA upregulated in GC and associated with patient overall survival.

### 3.2 LncSLCO1C1 promotes tumour progression by enhancing proliferation and metastasis of GC cells

**FIGURE 2:**
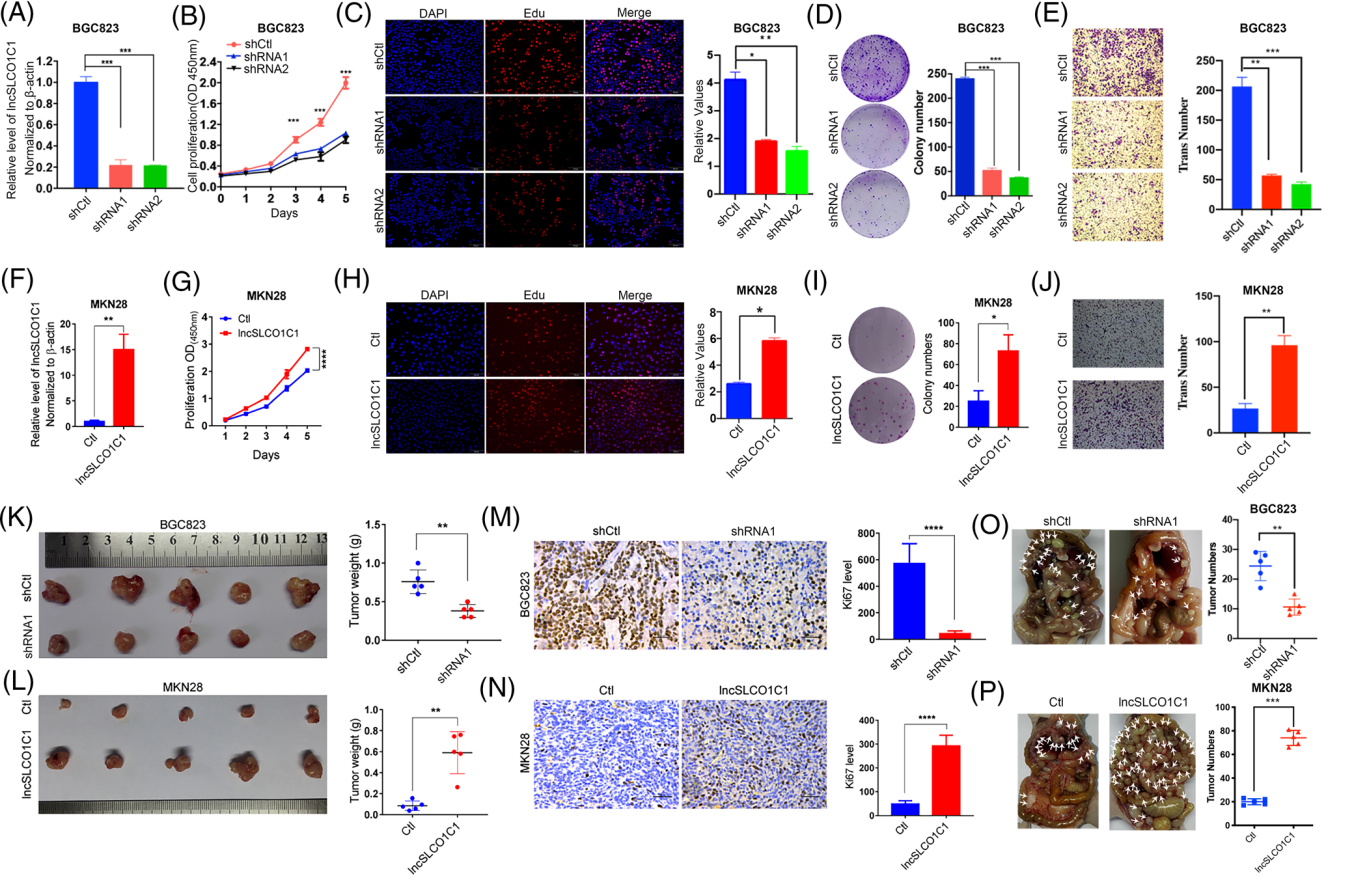
lncSLCO1C1 promotes tumour growth by enhancing cell proliferation and DNA repair. (A and F) The expression of lncSLCO1C1 in BGC823 and MKN28 cells transfected with negative control, shSLCO1C1 and lncSLCO1C1 vectors, respectively, as detected by quantitative real‐time polymerase chain reaction (qRT‐PCR). β‐Actin served as the internal reference. (B and G) Proliferation of BGC823 and MKN28 cells, where the expression of lncSLCO1C1 was modified, as measured using a CCK‐8 kit. (C and H) Red fluorescence generated by EDU staining shows the status of DNA replication in BGC823 and MKN28 cells where the expression of lncSLCO1C1 was modified. DAPI stains the cell nucleus. Bars show the intensity of red fluorescence, which was statistically calculated based on five slices. (D and I) Colony formation of BGC823 and MKN28 cells where the expression of lncSLCO1C1 was modified. Bars show colony numbers statistically calculated based on three wells. (E and J) Transwell assays applied to detect the migration ability of BGC823 and MKN28. Bars show the number of cells through the membrane statistically calculated based on three wells. (K and L) Xenografts from nude mice whose axillae were injected with BGC823 or MKN28 cells where the expression of lncSLCO1C1 was stably modified. Scatter plots showing the tumour weights. (M and N) Immunohistochemistry (IHC) staining for tumour cell proliferation with Ki67 antibody. Bars show the level of Ki67, which was statistically calculated based on five tumours. (O and P) Peritoneal metastasis models exhibited the metastatic ability of gastric carcinoma (GC) cells. White arrow pointed the metastatic site. Scatter plots showing the metastatic tumour number. In all figures, data are presented as mean ± standard error of the mean (SEM). The asterisks represented the statistical *p*‐value (*p < .05; **p < .01; ***p < .001; ****p < .0001; Student's test)

To explore the role of lncSLCO1C1 in GC progression, the expression of lncSLCO1C1 in six GC cell lines was measured. LncSLCO1C1 expression was higher in HGC27, SGC7901, BGC823 and MKN45 cells compared to that of adjacent non‐tumour tissue, but was almost undetectable in MKN74 and MKN28 cell lines as well as in GES‐1 (Figure S2A). We then downregulated the expression of lncSLCO1C1 in both BGC823 (Figure 2A) and SGC7901 (Figure S2B) cell lines with specific shRNAs delivered by lentiviral transductions and upregulated the expression of lncSLCO1C1 in MKN28 cells (Figure 2F) by transfection of full‐length lncSLCO1C1‐encoded expression vector.

We noticed that downregulation of lncSLCO1C1 expression by shRNAs against lncSLCO1C1 also resulted in reduction of PDE3A expression as the shRNAs recognised both sequences (see Figure S2B), whereas PDE3A siRNAs (Data S4) downregulated PDE3A mRNA expression much more than it did in lncSLCO1C1 expression (Figure S2C), suggesting that lncSLCO1C1 shRNAs can influence both lncSLCO1C1 and PDE3A mRNA whilst PDE3A siRNAs can only affect PDE3A mRNA. Primers against lncSLCO1C1 detect both lncSLCO1C1 and PDE3A mRNA whilst the PDE3A primer can only recognise PDE3A itself. Therefore, we investigated if lncSLCO1C1 expression would affect PDE3A expression in GC cells. Upregulation of lncSLCO1C1 did not influence PDE3A expression in MKN28 cells (Figure S2D,E).

Next, we evaluated the distribution of lncSLCO1C1 in GC cells. qRT‐PCR showed that approximately 70% lncSLCO1C1 is distributed in nucleus, whilst ∼30% existed in cytoplasm (Figure S2F,G). FISH assays confirmed the existence of high abundance of lncSLCO1C1 in GC cell nucleus (Figure S2H), whilst PDE3A mainly located in the cytoplasm (Figure S2I). Downregulation of lncSLCO1C1 in both nucleus and cytoplasm by lncSLCO1C1 shRNAs (Figure S2J,K).

Because lncSLCO1C1 is strongly associated with tumour size (see Figure 1F,I), we investigated if lncSLCO1C1 promotes cell survival ability in GC. In vitro experiments showed lncSLCO1C1 downregulation significantly inhibits proliferation and colony formation of BGC823 and SGC7901 cells (Figures 2B–D and S3A–C) whilst lncSLCO1C1 upregulation increases proliferation of MKN28 cells (Figure 2G–I). In contrast, both downregulation and upregulation of PDE3A did not affect proliferation and colony formation of these cell lines (Figures S3E–Q). Importantly, upregulation of PDE3A did not influence the colony formation of BGC823 and SGC7901 cells in which shSLCO1C1 has been downregulated (Figure S3R,S). In addition, downregulation of lncSLCO1C1 reduced the migration ability of GC cells (Figures 2E and S3D) whereas upregulation of lncSLCO1C1 increased the migration ability of GC cells (Figure 2J). In vivo xenograft experiments demonstrated that lncSLCO1C1 downregulation significantly decreases tumour growth of BGC823 and SGC7901 cells (Figures 2K and S3T) by inhibiting tumour cell proliferation (Figures 2M and S3U) whilst lncSLCO1C1 upregulation promotes tumour growth (Figure 2L,N). Similarly, investigation in peritoneal metastasis models revealed that upregulation of lncSLCO1C1 enhances metastasis of GC cells, and downregulation of lncSLCO1C1 produces an opposite effect (Figures 2O,P and S3V). Taken together, the results suggested that lncSLCO1C1 drive disease progression in vitro and in vivo by increasing the proliferation, migration and metastasis of GC cells.

### 3.3 LncSLCO1C1 prevents DNA damage of GC cells

**FIGURE 3:**
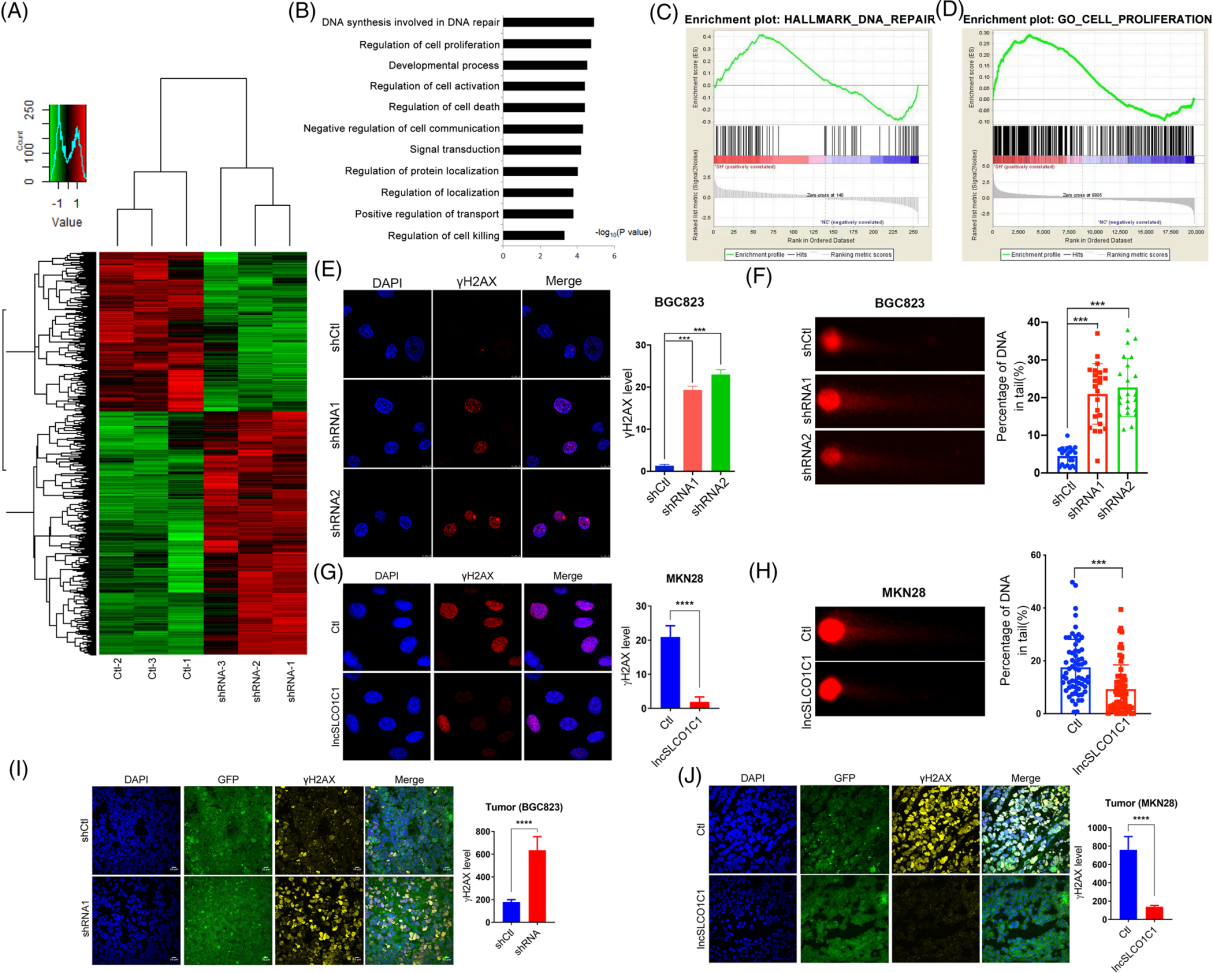
lncSLCO1C1 enhances DNA repair in vitro and in vivo. (A) A heat map showed aberrant expression of genes (*p *< .05) in BGC823 cells where lncSLCO1C1 was downregulated. (B) Gene ontology (GO) analysis shows that the cell proliferation and DNA repair are most involved in the regulation of lncSLCO1C1 in BGC823 cells. (C and D) Enrichment analysis shows that multiple genes associated with DNA repair and cell proliferation were invoked when lncSLCO1C1 was downregulated in BGC823 cells. (E and G) Red fluorescence shows the level of γH2AX in BGC823 and MKN28 cells where lncSLCO1C1 was knocked down or overexpressed, respectively. DAPI indicates the cell nucleus. Bars show the intensity of red fluorescence, which was statistically calculated based on five slices. The data are presented as mean ± standard error of the mean (SEM). The asterisks represented the statistical *p*‐value (*p < .05; **p < .01; ***p < .001; ****p < .0001; Student's test). (F and H) Comet assays were used to detect the effect of lncSLCO1C1 on DNA damage. Bars show the damaged DNA in the tail, which was statistically calculated based on three repeated biological experiments. The data are presented as mean ± SEM. The asterisks represented the statistical *p*‐value (*p < .05; **p < .01; ***p < .001; ****p < .0001; Student's test). (I and J) Yellow fluorescence shows the level of γH2AX in xenografts generated from BGC823 and MKN28 cells where the expression of lncSLCO1C1 was decreased or increased. GFP indicates the expression of shlncSLCO1C1 and lncSLCO1C1 vectors in BGC823 and MKN28 cells. DAPI indicates the cell nucleus. Bars show the intensity of yellow fluorescence, which was statistically calculated based on five slices. The data are presented as mean ± SEM. The asterisks represented the statistical *p*‐value (*p < .05; **p < .01; ***p < .001; ****p < .0001; Student's test)

To explore the underlying mechanism by which lncSLCO1C1 regulates tumour growth, we performed microarray analysis of BGC823 cells following lncSLCO1C1 downregulation (Data S5). A total of 1782 differentially expressed genes were obtained in lncSLCO1C1 downregulated cells (shRNA), compared to that of control cells (shCtl) (Figure 3A), of which 1075 were upregulated and 707 downregulated (Data S5). Pathway enrichment analysis of these differentially expressed genes showed lncSLCO1C1 downregulation interferes several cellular processes (Figure 3B), particularly those which regulate cell proliferation and DNA repair (Figure 3C,D). Further analysis showed that downregulation of lncSLCO1C1 reduced the expression of several cell cycle‐associated genes, whilst upregulation of lncSCLO1C1 had opposite results (Figure S4A). Downregulation of lncSLCO1C1 caused the cell cycle arrest at G1, whilst upregulation of lncSLCO1C1 in MKN28 cells accelerate the cell cycle (Figure S4B). These results suggest the promotion of tumour progression by lncSLCO1C1 may be conferred by increasing cell proliferation and preventing DNA damage of tumour cells.

Since γH2AX is generally considered a hallmark of DNA damage, we examined DNA repair in GC cells by measuring the expression of γH2AX. Immunofluorescent staining showed that downregulation of lncSLCO1C1 significantly increased the level of γH2AX in BGC823 and SGC7901 cells in vitro (Figures 3E and S4C) and in xenograft tissues in vivo (Figures 3I and S4E). Comet assays substantiated the results that downregulation of lncSLCO1C1 enhanced DNA damage (Figures 3F and S4D). Clearly, downregulation of PDE3A did not affect the expression of γH2AX in these cell lines at all (Figures S4F,G). In agreement with the downregulation, upregulation of lncSLCO1C1 decreased the level of γH2AX in vitro and in vivo (Figure 3G,J) and reduced DNA damage of MKN28 cells in comet assays (Figure 3H). Therefore, we concluded that lncSLCO1C1 serve as an oncogene to increase the survival ability of GC cells by preventing DNA damage in tumour cells.

### 3.4 LncSLCO1C1 serves as a scaffold to bind with SSRP1, H2A and H2B in nucleus

**FIGURE 4:**
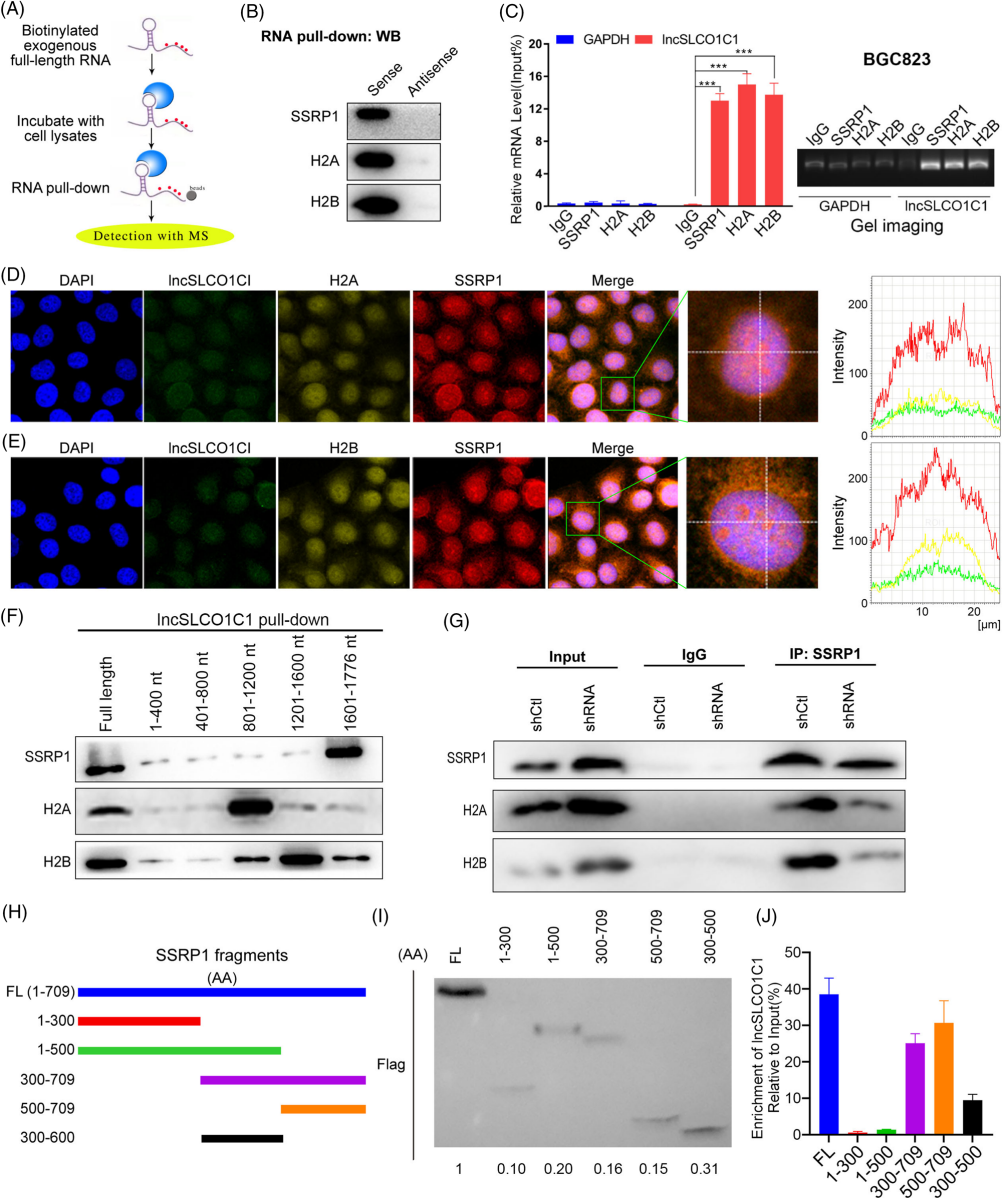
lncSLCO1C1 interacts with structure‐specific recognition protein 1 (SSRP1), H2A and H2B in nucleus. (A) A schematic diagram showing the RNA pull‐down experimental procedure. (B) RNA pull‐down followed by Western blotting shows SSRP1, H2A and H2B in the complex that was pulled down from BGC823 cells by biotin‐labelled lncSLCO1C1. Sense indicates using the full‐length sequence of lncSLCO1C1. Antisense sequence of lncSLCO1C1 was used as a control. (C) Graph showing enrichment of lncSLCO1C1 in the complex that was isolated from BGC823 cells using anti‐SSRP1, anti‐H2A or anti‐H2B antibodies via RNA immunoprecipitation (RIP) assays. The data are presented as mean ± standard error of the mean (SEM). The asterisks represented the statistical *p*‐value (*p < .05; **p < .01; ***p < .001; ****p < .0001; whilst one‐way analysis of variance (ANOVA) test). (D and E) Green fluorescence indicating the level and distribution of lncSLCO1C1 in BGC823 cells. Yellow fluorescence shows the level and distribution of H2A and H2B. Red fluorescence shows the level and distribution of SSRP1 in BGC823 cells. DAPI indicates the cell nucleus. The fluorescence intensity of the three colours was analysed using software. (F) Western blotting shows SSRP1, H2A and H2B in the complex isolated from BGC823 cells using different fragments of the lncSLCO1C1 sequence. (G) For Western blotting in the input group, the expression of SSRP1, H2A and H2B was adjusted to the same level for a visual comparison to the levels of the IP:SSRP1 group. In the IP:SSRP1 group, Western blotting shows the levels of the three proteins in the complex isolated from the lysate (the concentration was adjusted based on that of the input group) using anti‐SSRP1 antibody. (H) Schematic of truncated SSRP1 proteins. (I) Western blot of truncated SSRP1 proteins. (J) Detailed RIP exhibited the binding ability of truncated SSRP1 proteins with lncSLCO1C1. The data are presented as mean ± SEM. The asterisks represented the statistical *p*‐value (*p < .05; **p < .01; ***p < .001; ****p < .0001; whilst ANOVA test)

To dissect the molecular mechanism by which lncSLCO1C1 prevents DNA damage in GC cells, RNA pull‐down assays combined with mass spectrometry analysis (MS) in BGC823 and SGC7901 cells were performed (Figure 4A). At least 156 and 36 proteins were revealed to potentially interact with lncSLCO1C1 in BGC823 and SGC7901, respectively (http://www.pantherdb.org/), 25%–50% of which would bind nucleotides (Figure S5A,B; Data S6). Of these proteins, only SSRP1, H2A and H2B were overlapped in both cell lines. Both literature and our experiment showed that SSRP1 could bind with H2A/H2B (Figure S5C), we wounded what is the role of lncSLCO1C1 in this complex. We demonstrated the interaction between lncSLCO1C1 and SSRP1, H2A or H2B by RNA pull‐down followed by WB and RIP followed by qRT‐PCR experiments (Figures 4B,C and S5D–F). Triple‐labelling immunofluorescence followed by laser scanning confocal microscopy (LSCM) experiments showed lncSLCO1C1 (green), H2A or H2B (yellow) and SSRP1 (red) overlapped predominantly in nucleus of BGC823 cells (Figure 4D,E). Therefore, our results suggest the SSRP1/H2A/H2B complex contains at least another member, lncSLCO1C1.

Next, we investigated the potential binding sites of lncSLCO1C1 with SSRP1, H2A or H2B. We synthesised five fragments covering the full‐length of lncSLCO1C1 and performed RNA pull‐down combined with WB assays (Figure 4F). The results showed that the 1601–1776 nt fragment interacts strongest with SSRP1; the 801–1200 nt fragment with H2A and the 1201–1600 nt fragment with H2B (Figure 4F). In addition, bioinformatics analysis (http://service.tartaglialab.com/page/catrapid_group) also suggested the possible interactive sites of lncSLCO1C1 with SSRP1, H2A and H2B (Figure S5G), and such interactions are clearly visualised on predictive 3D models (Figure S5H) (http://genesilico.pl/NPDock). To dissect the role of lncSLCO1C1 in the complex, we performed Co‐IP assays of SSRP1 with H2A or H2B upon downregulation of lncSLCO1C1 in BGC823 cells (Figure 4G). lncSLCO1C1 downregulation significantly reduced the amount of both H2A and H2B that were pulled down by anti‐SSRP1 antibody, suggesting lncSLCO1C1 facilitate the interaction amongst SSRP1, H2A and H2B, and act as a scaffold molecule to enhance the function of the SSRP1/H2A/H2B complex in DNA damage repair.

In addition, we also explored the binding area of SSRP1 with lncSLCO1C1. Bioinformatic analysis revealed that lncSLCO1C1 binds the tail region of SSRP1 (see Figure S5H). By constructed five truncated fragments of SSRP1 for detailed RIP analysis (Figure 4H,I), we demonstrated that tail region (500‐709) of SSRP1 binds with lncSLCO1C1, whilst deletion of tail region (500‐709) greatly reduces the binding ability (Figure 4J). Collectively, our findings demonstrated that lncSLCO1C1 interacts with 500‐709 aa region of SSRP1 through its 1601–1776 nt.

### 3.5 LncSLCO1C1 mediates tumour resistance to oxaliplatin by enhancing SSRP1‐induced DNA repair

**FIGURE 5:**
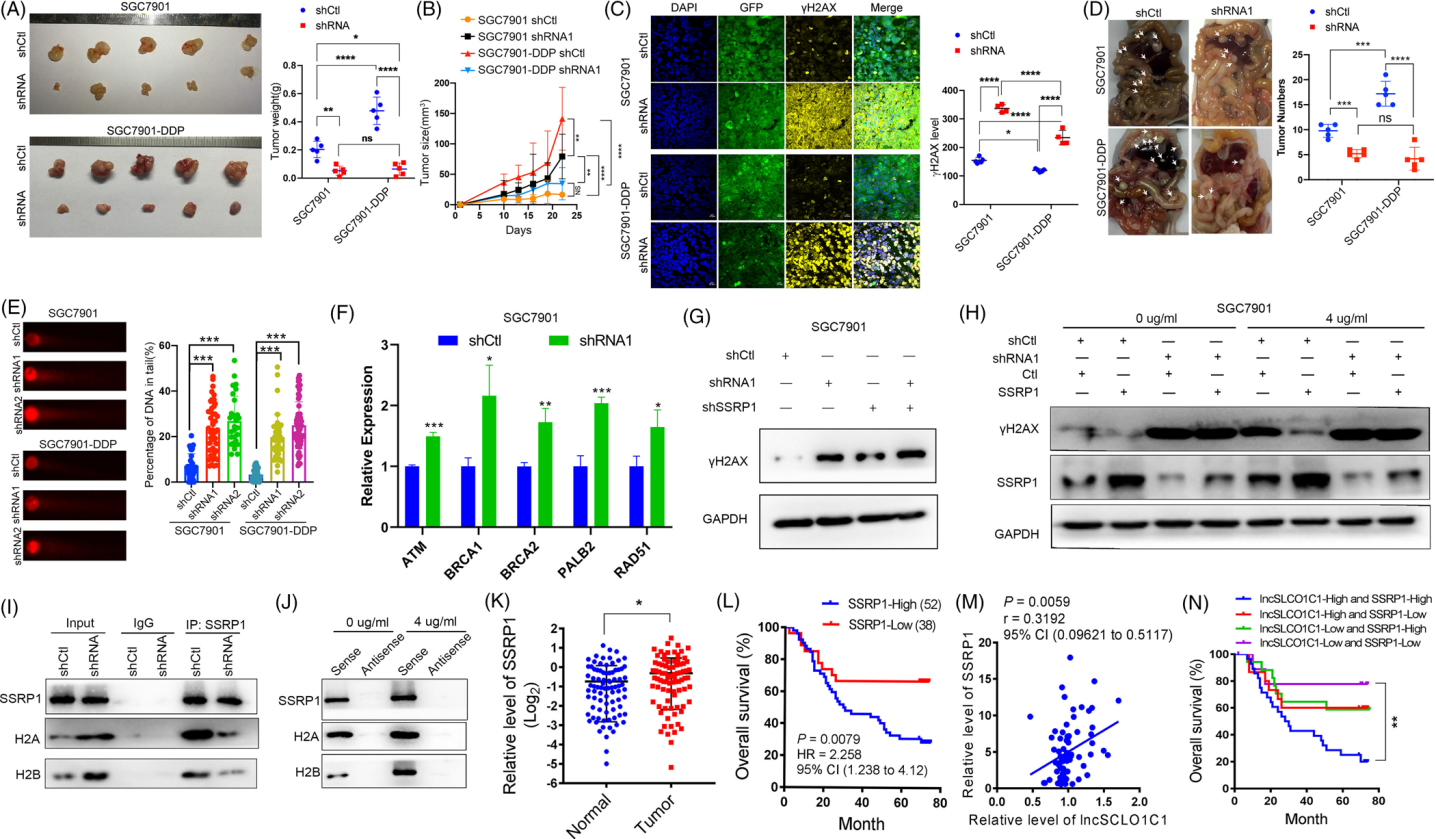
lncSLCO1C1 mediates tumour resistance to oxaliplatin by enhancing structure‐specific recognition protein 1 (SSRP1)‐induced DNA repair. (A) Xenografts from nude mice whose axillae were injected with SGC7901 or SGC7901‐DDP cells and whose enterocoelias were injected with oxaliplatin every three days. Scatter plots showing tumour weights. The data are presented as mean ± standard error of the mean (SEM). The asterisks represented the statistical *p*‐value (*p < .05; **p < .01; ***p < .001; ****p < .0001; whilst one‐way analysis of variance (ANOVA) test). (B) Tumour growth curve after oxaliplatin treatment. Tumour volume was measured, *n* = 5. The data are presented as mean ± SEM. The asterisks represented the statistical *p*‐value (*p < .05; **p < .01; ***p < .001; ****p < .0001; whilst ANOVA test). (C) Yellow fluorescence shows the level of γH2AX in the xenografts. Green fluorescence indicates the expression of shCtl or shlncSLCO1C1 vectors in the SGC7901 or SGC7901‐DDP cells of the xenografts. DAPI indicates the cell nucleus. Each scatter plot shows the intensity of yellow fluorescence that was statistically calculated based on five slices within each xenograft. The data are presented as mean ± SEM. The asterisks represented the statistical *p*‐value (*p < .05; **p < .01; ***p < .001; ****p < .0001; whilst ANOVA test). (D) Peritoneal metastasis assays show the metastatic ability of SGC7901 and SGC7901‐DDP with oxaliplatin treatment (5 mg/kg). Scatter plots showing the metastatic tumour number. The data are presented as mean ± SEM. The asterisks represented the statistical *p*‐value (*p < .05; **p < .01; ***p < .001; ****p < .0001; whilst ANOVA test). (E) Comet assays show the amount of damaged DNA in gastric carcinoma (GC) cells with oxaliplatin treatment (4 μg/ml), where lncSLCO1C1 was downregulated. Bars show the percentage of DNA in tail, which was statistically calculated based on three repeated biological experiments. The data are presented as mean ± SEM. The asterisks represented the statistical *p*‐value (*p < .05; **p < .01; ***p < .001; ****p < .0001; whilst ANOVA test). (F) Quantitative real‐time polymerase chain reaction (qRT‐PCR) shows the expression of DNA repair‐related genes after lncSLCO1C1 was suppressed. (G) Western blotting shows the expression of levels of γH2AX when lncSLCO1C1 or/and SSRP1 is suppressed. (H) Western blotting shows the levels of γH2AX, SSRP1 and GAPDH (as an internal reference) in SGC7901 cells that were treated with or without oxaliplatin (4 μg/ml), where lncSLCO1C1 was knocked down and/or SSRP1 was overexpressed using the corresponding vectors. (I) Under the oxaliplatin treatment, the bind relationship between SSRP1 and H2A/H2B were detected by co‐immunoprecipitation (Co‐IP) assay. (J) The binding relationship of lncSLCO1C1 and SSRP1/H2A/H2B was detected by RNA pull‐down and Western blot. (K) Scatter plots showing the expression of SSRP1 protein in Cohort 2 (*n* = 90), detected using immunohistochemistry. The data are presented as mean ± SEM. The asterisks represented the statistical *p*‐value (*p < .05; **p < .01; ***p < .001; ****p < .0001; Student's test). (L) Overall survival analysis shows the survival time of GC patients with high or low expression of SSRP1 in Cohort 2 (*n* = 90). High expression indicates that the expression in GC tissues divided by that in the adjacent normal tissues is more than 1 (*p* = .0079; log‐rank test). (M) Scatter plots showing the correlation between the expression of SSRP1 protein and lncSLCO1C1 in Cohort 2 (*n* = 90) (*p* = .0059; Pearson correlation analysis). (N) Overall survival analysis shows the survival time of GC patients with a high expression of both lncSLCO1C1 and SSRP1, a low expression of both lncSLCO1C1 and SSRP1, or one low and one high expression of either of the two in Cohort 2 (*n* = 90). The definition of high expression of lncSLCO1C1 or SSRP1 is the same as that described in (L). The asterisks represented the statistical *p*‐value (*p < .05; **p < .01; ***p < .001; ****p < .0001; log‐rank test)

Since SSRP1 is involved in platinum compound‐induced DNA repair and promotes chemotherapy resistance,^11^ we assessed if lncSLCO1C1 regulates tumour response to oxaliplatin chemotherapy. In xenograft mouse models (Figure 5A,B), SGC7901‐DDP cells grew much rapidly than SGC7901 cells upon oxaliplatin (1 μg/kg) treatment (SGC7901‐DDP shCtl vs. SGC7901 shCtl, *p *< .0001), confirming the drug resistance of SGC7901‐DDP.^23^ However, following lncSLCO1C1 downregulation, such an increased tumour growth was abolished upon oxaliplatin treatment (SGC7901‐DDP shRNA vs. SGC7901 shRNA, *p *> .05), suggesting lncSLCO1C1 mediate GC resistance to chemotherapy. Immunofluorescence analysis demonstrated the expression of γH2AX in xenograft sections is significantly reduced in SGC7901‐DDP tumour compared to SGC7901 tumour following oxaliplatin treatment (SGC7901‐DDP shCtl vs. SGC7901 shCtl, *p *< .05). Whereas lncSLCO1C1 downregulation significantly increased the expression of γH2AX in both xenograft tumours upon oxaliplatin treatment (shRNA vs. shCtl, both *p *< .0001), although γH2AX was higher in SGC7901 tumour than SGC7901‐DDP tumour (Figure 5C). This suggests the mediation of oxaliplatin resistance by lncSLCO1C1 is closely associated with regulation of DNA repair by lncSLCO1C1 via the lncSLCO1C1‐SSRP1/H2A/H2B complex. In peritoneal metastasis models, we demonstrated that downregulation of lncSLCO1C1 reduces tumour metastasis of SGC7901‐DDP upon the treatment with oxaliplatin (Figure 5D). In addition, comet assays revealed that downregulation of lncSLCO1C1 increases the DNA damage in SGC7901 and SGC7901‐DDP cells (Figure 5E). In addition, downregulation of lncSLCO1C1 increased the expression of key DNA damage repair genes (Figure 5F), suggesting that lncSLCO1C1 regulate the DNA damage repair. Indeed, downregulation of lncSLCO1C1 resulted in heavier DNA damage than SSRP1 downregulation (Figure 5G). To further investigate if lncSLCO1C1 in the complex controls DNA damage repair, we modulated the expression of lncSLCO1C1 and SSRP1 in SGC7901 cells by downregulating lncSLCO1C1 and upregulating SSPR1 (Figure 5H). In the absence of oxaliplatin, SSRP1 upregulation decreased γH2AX protein expression (Figure 5H). However, following lncSLCO1C1 downregulation, the expression of γH2AX was significantly increased; SSRP1 upregulation did not decrease the expression level of γH2AX (Figure 5H). Similar results were obtained upon oxaliplatin (4 μg/ml) treatment suggesting lncSLCO1C1 is fundamental for SSRP1‐induced DNA repair (Figure 5H). Interestingly, upon oxaliplatin treatment (4 μg/ml), downregulation of lncSLCO1C1 reduced the amount of both H2A and H2B that were precipitated by specific anti‐SSRP1 antibody (Figure 5I); oxaliplatin treatment increased the amount of SSRP1, H2A and H2B that were pulled down by lncSLCO1C1 (Figure 5J). Altogether, the results suggested lncSLCO1C1 act as a scaffold to enhance the stability and function of the SSRP1/H2A/H2B complex in DNA damage repair.

To investigate the clinical relevance of aberrant lncSLCO1CS and SSRP1 expression on patient overall survival, we performed anti‐SSRP1 immunohistochemistry staining of clinical samples in Cohort 2. The expression of SSRP1 was significantly increased in GC (Figure 5K). Patients with high SSRP1 level have poor overall survival (Figure 5L). A positive correlation was found between the expression of SSRP1 and lncSLCO1C1 (Figure 5M). Interestingly, overall survival analysis combining the expression of lncSLCO1C1 and SSRP1 showed that patients with high expression of both lncSLCO1C1 and SSRP1 have the worst overall survival, whilst patients with low expression of both lncSLCO1C1 and SSRP1 have increased overall survival (Figure 5N).

Taken together, we concluded that lncSLCO1C1 mediate tumour resistance to chemotherapy with oxaliplatin by enhancing SSRP1‐induced DNA repair in GC cells, resulting in poor patient overall survival.

### 3.6 LncSLCO1C1 increases SSRP1 expression by adsorbing miR‐211‐5p and miR‐204‐5p in cytoplasm

Oxaliplatin treatment significantly increased the level of lncSLCO1C1 and SSRP1 in a dose‐dependent manner as revealed by FISH, qRT‐PCR and WB (Figure S6A–C). However, downregulation of lncSLCO1C1 significantly reduced SSRP1 at mRNA and protein level, abolishing the effect of oxaliplatin (Figure S6D,E) suggesting lncSLCO1C1 mediates the increased expression of SSRP1 by oxaliplatin. In contrast, downregulation of SSRP1 could not affect the level of lncSLCO1C1 (Figure S6F,G), suggesting lncSLCO1C1 be an upstream regulator of SSRP1.

**FIGURE 6:**
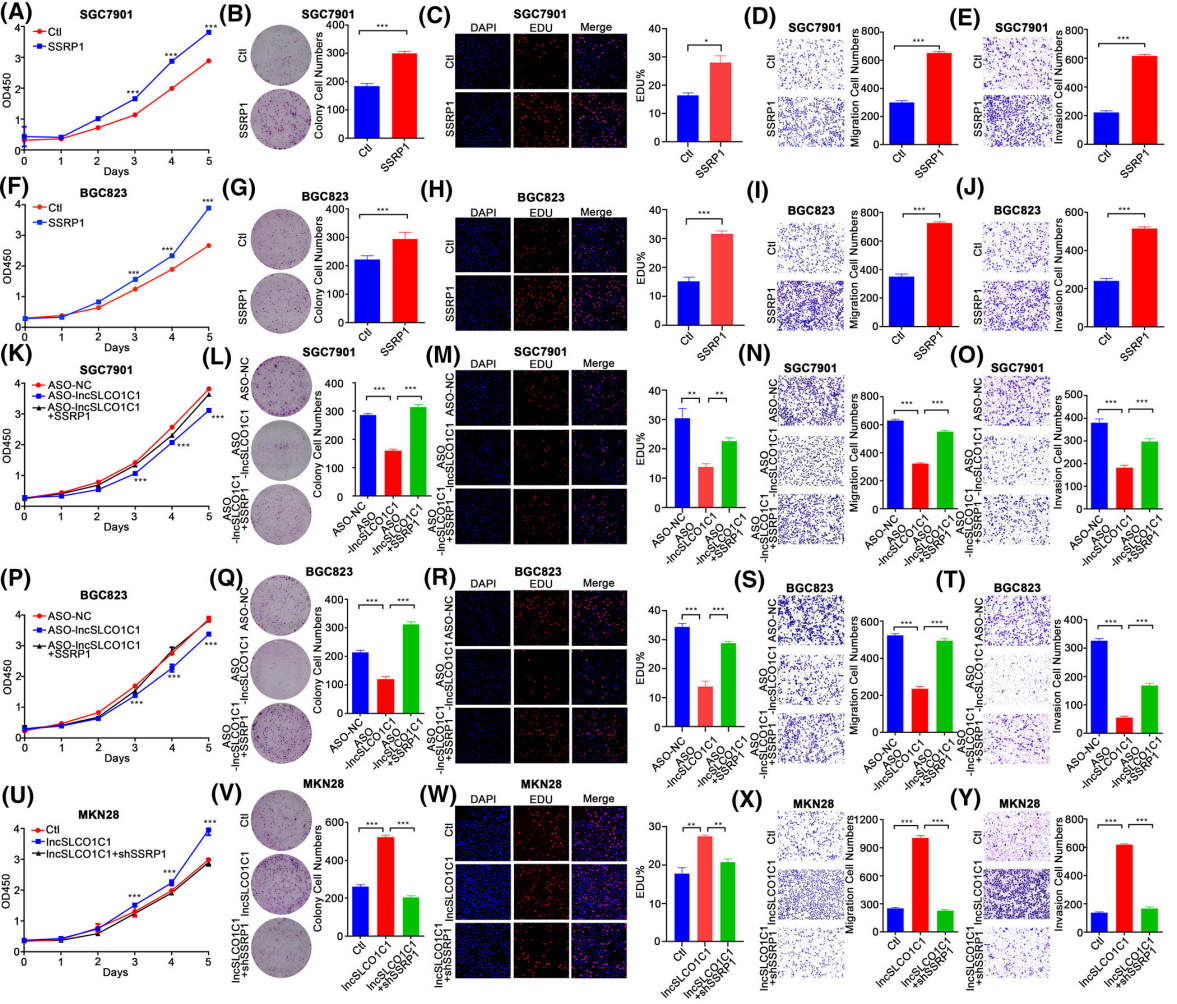
lncSLCO1C1 promotes proliferation and metastasis of gastric carcinoma (GC) cells via structure‐specific recognition protein 1 (SSRP1). (A and F) CCK‐8 assay was used to detect the proliferation ability of GC cells when SSRP1 was overexpressed in SGC7901 (A) and BGC823 (F). (B and G) Colony assay was used to detect the proliferation ability of GC cells when SSRP1 was overexpressed in SGC7901 (B) and BGC823 (G). (C and H) EDU assay was used to detect the proliferation ability of GC cells when SSRP1 was overexpressed in SGC7901 (C) and BGC823 (H). (D and I) Transwell assay was used to detect the migration ability of GC cells when SSRP1 was overexpressed in SGC7901 (D) and BGC823 (I). (E and J) The invasion ability of GC cells was determined by Matrigel Transwell assay, when SSRP1 was overexpressed in SGC7901 (E) and BGC823 (J). (K and P) The proliferation ability was detected in SGC7901 (K) and BGC823 (P) by CCK‐8 assay, when lncSLCO1C1 suppression or lncSLCO1C1 suppression but SSRP1 overexpression. (L and Q) The proliferation ability was detected in SGC7901 (L) and BGC823 (Q) by Colony assay, when lncSLCO1C1 suppression or lncSLCO1C1 suppression but SSRP1 overexpression. (M and R) The proliferation ability was detected in SGC7901 (M) and BGC823 (R) by EDU assay, when lncSLCO1C1 suppression or lncSLCO1C1 suppression but SSRP1 overexpression. (N and S) The migration ability was detected in SGC7901 (N) and BGC823 (S) by Transwell assay, when lncSLCO1C1 suppression or lncSLCO1C1 suppression but SSRP1 overexpression. (O and T) The invasion ability was detected in SGC7901 (O) and BGC823 (T) by Matrigel Transwell assay, when lncSLCO1C1 suppression or lncSLCO1C1 suppression but SSRP1 overexpression. (U–W) The proliferation ability was detected in MKN28 by CCK‐8 assay, Colony assay and EDU assay, when lncSLCO1C1 overexpression or lncSLCO1C1 overexpression but SSRP1 suppression. (X and Y) The metastasis of MKN28 cells was detected by Transwell assay (X) and Matrigel Transwell assay (Y), when lncSLCO1C1 overexpression or lncSLCO1C1 overexpression but SSRP1 suppression. In all figures, data are presented as mean ± standard error of the mean (SEM). The asterisks represented the statistical *p*‐value (*p < .05; **p < .01; ***p < .001; ****p < .0001; Student's test)

We demonstrated upregulation of SSRP1 promotes cell proliferation, migration and invasion of both SGC7901 (Figure 6A–E) and BGC823 (Figure 6F–J). Downregulation of lncSLCO1C1 by ASO reduced cell proliferation, migration and invasion of both SGC7901 (Figure 6K–O) and BGC823 (Figure 6P–T); all these effects conferred by lncSLCO1C1 downregulation were able to be rescued by SSRP1 upregulation (see Figure 6K–T). However, downregulation of SSRP1 significantly reduced GC progression enhanced by upregulation of lncSLCO1C1 in MKN28 cells (Figure 6U–Y). Collectively, these results suggested that lncSLCO1C1 promote GC progression by SSRP1.

**FIGURE 7:**
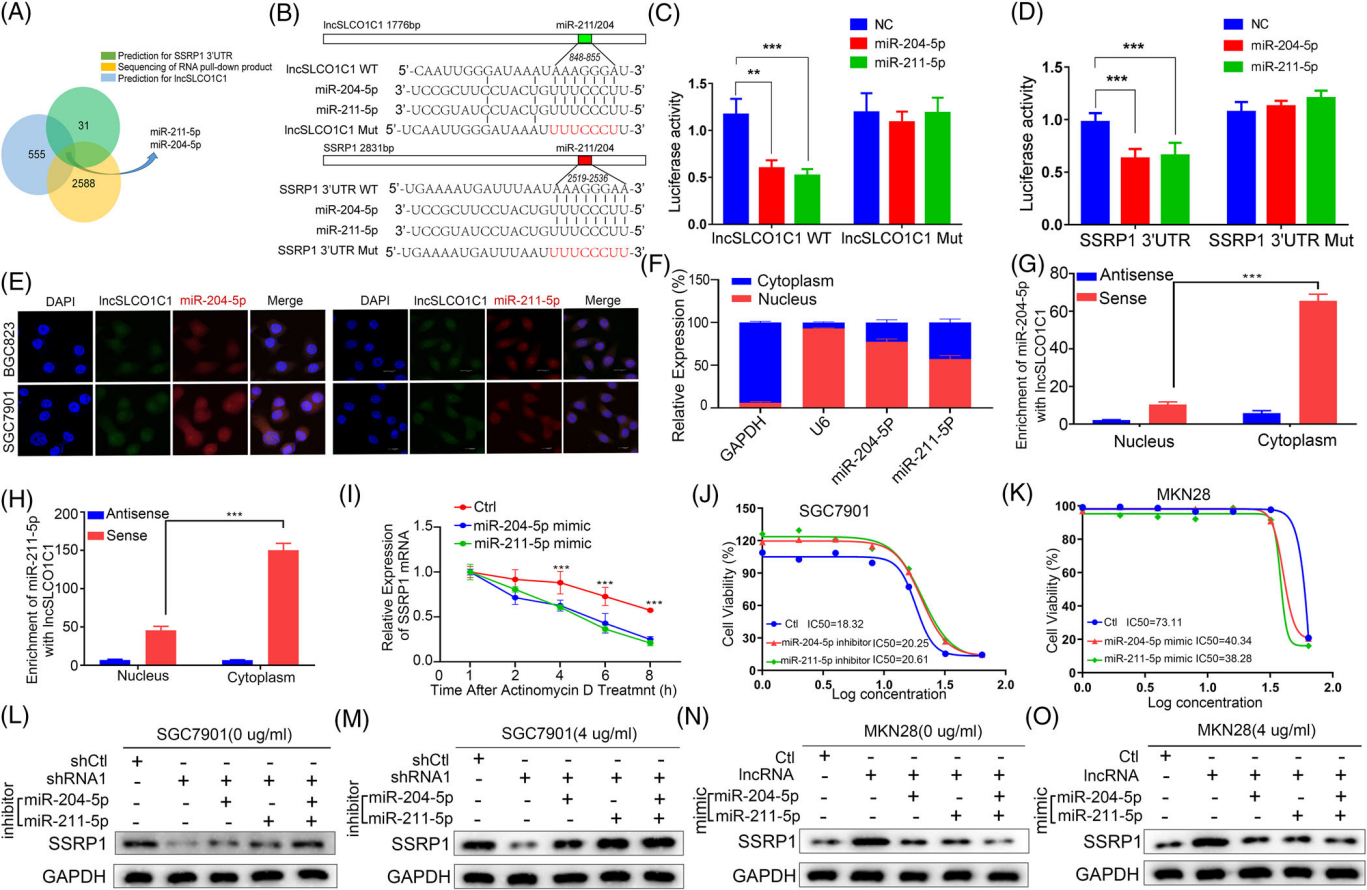
lncSLCO1C1 increases structure‐specific recognition protein 1 (SSRP1) expression by adsorbing miR‐211‐5p and miR‐204‐5p. (A) Integration of analyses of data from three aspects, including the prediction of the binding site between miRNAs and lncSLCO1C1 or SSRP1 and the sequencing of RNA pull‐down products. (B) The sequence of the binding site between miR‐204‐5p/miR‐211‐5p and lncSLCO1C1 or SSRP1. Red‐labelled sequences indicate the design of the mutant site. (C and D) Graphs show luciferase activity in HEK293 cells where luciferase reporter vectors containing lncSLCO1C1, lncSLCO1C1 mutant, SSRP1 3′UTR or SSRP1 3′UTR mutant, and miR‐204‐5p or miR‐211‐5p were transfected. The data are presented as mean ± standard error of the mean (SEM). The asterisks represented the statistical *p*‐value (*p < .05; **p < .01; ***p < .001; ****p < .0001; whilst one‐way analysis of variance (ANOVA) test). (E) Green fluorescence reflects the level and distribution of lncSLCO1C1 in BGC823 and SGC7901 cells. Red fluorescence shows the level and distribution of miR‐204‐5p or miR‐211‐5p in BGC823 and SGC7901 cells. DAPI indicates the cell nucleus. (F) The location of miR‐211‐5p and miR‐204‐4p was detected by Nucleocytoplasmic fractionation assay and quantitative real‐time polymerase chain reaction (qRT‐PCR) assay. (G and H) The binding relationship between lncSLCO1C1 and miR‐204‐5p (G)/miR‐211‐5p (H) were detected by Nucleocytoplasmic fractionation assay and RNA–RNA immunoprecipitation (IP)‐PCR. The data are presented as mean ± SEM. The asterisks represented the statistical *p*‐value (*p < .05; **p < .01; ***p < .001; ****p < .0001; Student's test). (I) The stability of SSRP1 mRNA was detected after Actinomycin D was added. The data are presented as mean ± SEM. The asterisks represented the statistical *p*‐value (*p < .05; **p < .01; ***p < .001; ****p < .0001; whilst ANOVA test). (J and K) The sensitivity of oxaliplatin was detected when inhibitor (J) or mimics (K) were added into target cells. (L–O) Western blotting shows the expression of SSRP1 and GAPDH (as an internal reference) in MKN28 and SGC7901 cells where lncSLCO1C1, miR‐204‐5p and miR‐211‐5p were expressed or knocked down, with or without oxaliplatin treatment (4 μg/ml), respectively

As lncSLCO1C1 regulates the mRNA expression of SSRP1, we hypothesised that lncSLCO1C1 may serve as a ceRNA to adsorb some miRNAs that regulate SSRP1 expression. We first performed bioinformatics analysis and a total 555 and 31 miRNAs were predicted to bind tolncSLCO1C1 and the 3′ UTR of SSRP1, respectively (Data S1). We then sequenced the RNA products that were pulled down by lncSLCO1C1. Note that 2588 miRNAs were found to potentially bind lncSLCOC1 (Data S1). Cross‐intersection analysis showed that only miR‐211‐5p and miR‐204‐5p exist in all three sets of miRNAs (Figure 7A). Luciferase reporter experiments demonstrated that either miR‐204‐5p or miR‐211‐5p significantly inhibits the luciferase activity of wild‐type lncSLCO1C1 or 3′UTR of SSRP1, but not the corresponding control reporter that the binding site was mutated (Figure 7B–D). FISH assays showed that these two miRNAs were co‐localised with lncSLCO1C1 in both cytoplasm and nuclear of SGC7901 and BGC823 cells (Figure 7E). Nucleocytoplasmic fractionation assay and RNA–RNA IP assay (Figure 7F–H) revealed that the interaction of lncSLCO1C1 and miRNA‐211‐5p and miRNA‐204‐5p occurred predominantly in cytoplasm (see Figure 7G,H). The copy number of lncSLCO1C1, miR‐204‐5p or miR‐211‐5p was measured by qRT‐PCR (Figure S6H). In addition, we found that miR‐211‐5p and miR‐204‐5p could reduce the stability of SSRP1 mRNA (Figure 7I), suggesting that lncSLCO1C1, as a ceRNA, efficiently absorb these two miRNAs and thus increase SSRP1 expression. No significant difference was found for these two miRNAs when treated with oxaliplatin (Figure S6I). Addition of miRNA‐204‐5p (or miRNA‐211‐5p) mimics enhanced the DNA damage of GC cells (Figures S6J,K and 7K), whilst addition of miRNA‐204‐5P or miRNA‐211‐5P inhibitors reduced the DNA damage of GC cell (Figure 7J) WB showed that the expression of SSRP1 decreased by downregulation of lncSLCOC1 was recovered by transfection of either miRNA‐204‐5p inhibitors or miRNA‐211‐5p inhibitors into SGC7901 cells, without or with oxaliplatin treatment (Figure 7L,M). In contrast, the expression of SSRP1 increased by upregulation of lncSLCOC1 was abolished by transfection of either miRNA‐204‐5p mimics or miRNA‐211‐5p mimics into MKN28 cells, without or with oxaliplatin treatment (Figure 7N,O). Altogether, these results suggest lncSLCO1C1 act as a ceRNA molecular sponge to adsorb miRNA‐204‐5p and miRNA‐211‐5p and consequently increase the expression of SSRP1.

We further explore the role of lncSLCO1C1/miR‐204‐5p/miR‐211‐5p in GC progression. By qRT‐PCR, we demonstrated downregulation of lncSLCO1C1 significantly reduced the expression of FOXC1 and AP1S2 (Figure S6L), all of which are downstream targets of miR‐211‐5p/miR‐204‐5p.^24^, ^25^ In addition, in vitro experiments showed upregulation by transfection of either miR‐211‐5p mimic or miR‐204‐5p mimic significantly reduced cell proliferation and metastasis of SGC7901 (Figure S7A–E) and BGC823 (Figure S7F–J). Downregulation of either miR‐211‐5p or miR‐204‐5p abolished the effect on decreased cell proliferation, migration and invasion of SGC7901 (Figure S7K–M) and BGC823 (Figure S7P–T) by downregulation of lncSLCO1C1 expression. Consistently, addition of miR‐211‐5p mimic or miR‐204‐5p mimic abolished the effect on increased proliferation, migration and invasion of MKN28 promoted by upregulation of lncSLCO1C1 (Figure S7U–Y). Altogether, these results suggested that lncSLCO1C1 enhance GC progression through miR‐211‐5p and miR‐204‐5p.

## 4 DISCUSSION

In the present study, we have identified a novel lncRNA with a length of 1776 nt from GC tissue, which we named lncSLCO1C1. Comprehensive bioinformatic analyses and expression vector transfection assays demonstrated lncSLCO1C1 does not encode any proteins in GC cells. Interestingly, lncSLCO1C1 expression is significantly upregulated in GC tissue versus adjacent non‐tumour tissue, and is closely associated with tumour differentiation, tumour size and poor overall survival of GC patients. However, LncSLCO1C1 completely overlaps with the sequence from 6200 nt to 7975 nt within 3′‐UTR of PDE3A mRNA. Although PDE3A is involved in the development of several cancer types but its role in GC is still unclear.^26^, ^27^ By qRT‐PCR with primers specific for PDE3A, we found there is no difference for the expression of PDE3A between GC tissues and matched normal tissues.

Recently, increasing evidence showed that lncRNA played important roles in tumourigenesis and progression of GC.^28^ gastric cancer metastasis associated long noncoding RNA (GMAN) was reported to promote the metastasis of GC cells via improving expression of ephrin A1 expression through competitively binding with GMAN‐AS.^29^ GClnc1 may recruit the WDR5 and KAT2A in the nuclear and modify the target genes expression in GC progression.^30^ Colorectal neoplasia differentially expressed lncRNA (CRNDE) was reported to bind with SRSF6 protein to reduce its protein stability, regulating the GC chemoresistance.^31^ However, the detail mechanism of lncRNA in GC progression remains unclear. To investigate the biological function of lncSLCO1C1 in GC development, we modulated the expression of lncSLCO1C1 in tumour cells by lentivirus‐transduced upregulation and shRNA‐mediated downregulation. We demonstrated that lncSLCO1C1 enhances the growth and metastasis of GC cells in vitro and in vivo, suggesting that lncSLCO1C1 could promote the GC progression. However, alternation of PDE3A expression did not affect the growth of GC cells. By analysing the expression and biological functions of lncSLCO1C1 and PDE3A, we believe that lncSLCO1C1 is independent upon PDE3A in GC progression.

To address the underlying mechanism on how lncSLCO1C1 promotes tumour growth, we performed microarray analysis of lncSLCO1C1 downregulated tumour cells and pathway enrichment for differentially expressed genes obtained from the microarray. We revealed that lncSLCO1C1 regulates many cellular processes, particularly those regulating cell proliferation and DNA damage. lncRNAs are known to exert their biological functions in several ways. The most common is via the interaction of lncRNA with protein(s) or miRNA(s). To explore the molecular mechanism by which lncSLCO1C1 reduces DNA damage, we performed biotinylated lncRNA pull‐down assays with two cell lines followed by MS analysis.^32^, ^33^ We revealed that lncSLCO1C1 interacts with SSRP1, H2A and H2B in the nuclei of both cell lines through different domains and verified the interaction by lncRNA pull‐down/WB, RIP/qRT‐PCR, co‐localisation staining, bioinformatics analysis and 3D molecular modelling. It is well known that SSRP1 interacts with H2A and H2B to form a complex that facilitates DNA repair.^10^, ^34^, ^35^, ^36^ Since downregulation of lncSLCO1C1 could reduce the expression of SSRP1, in order to detect the scaffold role of lncSLCO1C1 in SSRP1/H2A/H2B complex, we artificially increased the cell amount in the shRNA group during the Co‐IP assay, to achieve the same expression of SSRP1 in both groups. When the expression of SSRP1 is similar in two groups (in short, we use SSRP1 as a reference), the binding ability of SSRP1 with H2A/H2B could be able to compare. Through this setting, we showed lncSLCO1C1 downregulation significantly attenuates the interaction of SSRP1 with H2A and H2B, but dramatically increased the expression of γH2AX in GC cells even in the presence of additional SSRP1. Taken together, we concluded that lncSLCO1C1 serves as a scaffold to increase and/or stabilise the interaction of SSRP1 with H2A and H2B, consequently enhancing SSRP1‐mediated DNA repair. It is noted that some of previously identified lncRNAs may also act as scaffolds to cross‐link different proteins,^29^, ^37^, ^38^ and promote DNA repair by scaffolding Ku80 and DNA‐PKc.^39^ However, no lncRNA has been shown to interact with the SSRP1/H2A/H2B complex.

Previous studies have shown the SSRP1 complex can repair a single or double strand break of DNA,^9^, ^34^ and this function is closely involved in platinum resistance.^11^ Therefore, we investigated if lncSLCO1C1 plays a crucial role in regulation of tumour response to chemotherapy. Indeed, lncSLCO1C1 mediates tumour resistance to oxaliplatin as downregulation of lncSLCO1C1 re‐sensitised the platinum‐resistant tumour to fully response oxaliplatin treatment by reducing the interaction of SSRP1 with H2A/H2B and increasing the expression of γH2AX in tumour cells. Surprisingly, we also found oxaliplatin treatment significantly increases the expression of both lncSLCO1C1 and SSRP1 in a dose‐dependent manner. Downregulation of lncSLCO1C1 fully abolished SSRP1 expression increased by oxaliplatin, whilst downregulation of SSRP1 did not affect the expression of lncSLCO1C1, suggesting that lncSLCO1C1 act as an upstream regulator of SSRP1 and mediate the increased expression of SSRP1 by oxaliplatin. In GC clinical samples, we found patients with high expression of both lncSLCO1C1 and SSRP1 have a much shorter overall survival than those with low expressions of both, highlighting the importance and functional relevance of lncSLCO1C1 and SSRP1 in GC. Therefore, lncSLCO1C1 may act as a potential biomarker to predict the therapeutic effect of platinum compounds and/or as a new therapeutic target against platinum‐resistant GCs.

Interestingly, we found that SSRP1 is induced by oxaliplatin treatment, but this increase can be inhibited by lncSLCO1C1 downregulation, suggesting that SSRP1 is the downstream target for lncSLCO1C1. To further explore the detail mechanism by which lncSLCO1C1 mediates the function of oxaliplatin in increasing SSRP1 expression, we pulled down miRNAs from tumour cells by lncSLCO1C1 and obtained thousands of miRNAs that might interact with lncSLCO1C1 in GC cells. As lncRNAs can serve as ceRNAs to adsorb miRNAs and increase the expression of miRNA‐target genes.^40^ We focused on the miRNAs that could bridge lncSLCO1C1 together with SSRP1. By cross‐comparison of all miRNAs obtained from lncRNA pull‐down experiments with those obtained from bioinformatic predictions that potentially interact with lncSLCO1C1 and 3′‐UTR of SSRP1, respectively, we found miR‐204‐5p/miR‐211‐5p are the only two candidates. Subsequent experiments confirmed that these two miRNAs not only bind lncSLCO1C1 but also target the mRNA of SSRP1. Thus, lncSLCO1C1 serves as a ceRNA molecular sponge to adsorb these two miRNAs in the cytoplasm and increase the expression of SSRP1 in GC cells. In fact, these two miRNAs have been reported to act as tumour suppressors in several types of cancers including melanoma,^41^ breast cancer^42^ and GC.^43^

## 5 CONCLUSIONS

We have identified a novel lncRNA, called lncSLCO1C1. LncSLCO1C1 is significantly upregulated in GC tumour tissues and closely associated with overall survival of GC patients. Alterations of lncSLCO1C expression in tumour cells demonstrated lncLSCO1C1 functions as an oncogene to promote GC progression and mediate tumour resistance to oxaliplatin by enhancing cell survival ability. Mechanically, in the nucleus lncSLCO1C1 acts as a scaffold to cross‐link and stabilise the SSRP1/H2A/H2B complex, consequently reducing DNA damage; whilst in the cytoplasm, lncSLCO1C1 serves as a ceRNA molecular sponge to adsorb miR‐204‐5p/miR‐211‐5p, thus increasing the expression of SSRP1. Oxaliplatin treatment induces the expression of lncSLCO1C1 further, which in turn increases tumour resistance to chemotherapy in a feedforward manner. Our results provide new insights on gastric carcinogenesis and chemoresistance and rationale for improving therapeutic efficacy of platinum‐based chemotherapies and developing novel intervention strategies against GC.

## CONFLICT OF INTEREST

The authors declare no conflict of interests.

## EXPECTS DATA SHARING

The data that support the findings of this study are available from the corresponding author upon reasonable request.

## Supporting information



Figure S1. Corresponding to Figure 1. (A) The level of lncSLCO1C1 in TRNIC database. The data are presented as mean ± standard error of the mean (SEM). The asterisks represented the statistical *p*‐value (*p < .05; **p < .01; ***p < .001; ****p < .0001; Student's test). (B) The relation of lncSLCO1C1 and PDE3A in gene database. (C) The expression of PDE3A in Cohort 1 (*n* = 49). The data are presented as mean ± SEM (*p* = .7401; Student's test). (D) The expression of PDE3A in Cohort 2 by immunohistochemistry (IHC) (*n* = 90). The data are presented as mean ± SEM (the “ns” represents no significance; Student's test). (E) Sequencing results of 5′‐ RACE and 3′‐RACE experiments. (F) Flag expression was detected using Western blot experiment. Empty, PDE3A and lncSLCO1C1 denote pcDNA3.1 empty vector, pcDNA3.1‐PDE3A‐flag vector and pcDNA3.1‐lncSLCO1C1‐flag vector, respectively. (G) Receiver operating characteristic (ROC) curve showing the expression of lncSLCO1C1 in Cohort 1 (*n* = 49) (*p* = .0088; Student's test). (H) Representative image of the in situ hybridisation for lncSLCO1C1 in Cohort 2 (*n* = 90). (I) ROC curve showing the expression of lncSLCO1C1 in Cohort 2 (*n* = 90) (*p* = .0007; Student's test)

Figure S2. Corresponding to Figure 2. (A) Scatter plots show the expression of lncSLCO1C1 in 49 pairs of gastric carcinoma (GC) and adjacent normal tissues and different GC cell lines. β‐Actin served as the internal reference. **p < .01, adjacent normal tissues were listed as control sample. The data are presented as mean ± standard error of the mean (SEM). The asterisks represented the statistical *p*‐value (*p < .05; **p < .01; ***p < .001; ****p < .0001; whilst one‐way analysis of variance (ANOVA) test). (B) Bars show the expression of lncSLCO1C1 and PDE3A mRNA in SGC7901 cells which were transfected with sh‐lncSLCO1C1 vectors. β‐Actin served as the internal reference. The data are presented as mean ± SEM. The asterisks represented the statistical *p*‐value (*p < .05; **p < .01; ***p < .001; ****p < .0001; whilst ANOVA test). (C) The expression of lncSLCO1C1 and PDE3A whilst PDE3A was suppressed. The data are presented as mean ± SEM. The asterisks represented the statistical *p*‐value (*p < .05; **p < .01; ***p < .001; ****p < .0001; whilst ANOVA test). (D and E) The mRNA and protein level of PDE3A in MKN28 cells which were transfected with lncSLCO1C1‐overexpressing vectors. The data are presented as mean ± SEM. The asterisks represented the statistical *p*‐value (*p < .05; **p < .01; ***p < .001; ****p < .0001; Student's test). (F) Nuclear plasma separation assay was performed in BGC823 cells, and Western blot assay was applied to measure the quality. (G) Graphs showing the distribution of β‐actin, U6 and lncSLCO1C1 in BGC823 cells. (H and I) The location of lncSLCO1C1 and PDE3A detected by fluorescence in situ hybridisation (FISH). (J and K) The expression and location of lncSLCO1C1 detected by FISH, when shRNAs are applied in SGC7901 and BGC823. Red colour shows the level and location of lncSLCO1C1. Blue colour indicates the cell nucleus stained by DAPI. The fluorescence intensity was analysed using Image J software. The data are presented as mean ± SEM. The asterisks represented the statistical *p*‐value (*p < .05; **p < .01; ***p < .001; ****p < .0001; whilst ANOVA test).

Figure S3. lncSLCO1C1 promotes the proliferation of SGC7901 cells in vitro and in vivo. (A) Proliferation of SGC7901 cells where lncSLCO1C1 was downregulated as analysed using a CCK‐8 kit. (B) Red fluorescence generated by 5‐Ethynyl‐2′‐deoxyuridine (EDU) staining shows the status of DNA replication in SGC7901 cells where the expression of lncSLCO1C1 was modified. DAPI indicates the cell nucleus. Graphs show the intensity of the red fluorescence that was statistically calculated based on five slices. (C) Colony formation of SGC7901 cells where the expression of lncSLCO1C1 was modified. Bars show clone numbers that were statistically calculated based on three wells. (D) Transwell assay exhibited the migration ability of SGC7901 cells. Bars show the trans‐membraned cells. (E and F) The proliferation of SGC7901 and BGC823 cells where PDE3A was suppressed as analysed using CCK‐8 kit. (G and H) Colony formation of SGC7901 and BGC823 cells where PDE3A was suppressed. Bars show clone numbers that were statistically calculated based of three wells. (I–K) PDE3A mRNA expression was detected by quantitative real‐time polymerase chain reaction (qRT‐PCR) in SGC7901, BGC823 and MKN28 which were transfected with PDE3A‐overexpressing vectors. (L–N) Proliferation of SGC7901, BGC823 and MKN28 cells where PDE3A was upregulated, analysed using a CCK‐8 kit. (O–Q) Colony formation of SGC7901, BGC823 and MKN28 cells where the expression of PDE3A was upregulated. Bars show the clone number that was statistically calculated based on three wells. (R and S) Colony formation of BGC823 and SGC7901 cells where lncSLCO1C1 expression was suppressed, combined with or without an enforced expression of PDE3A. Bars show the clone number that was statistically calculated based on three wells. (T) Xenografts from nude mice whose axillae were injected with SGC7901 cells where the expression of lncSLCO1C1 was stably modified. Scatter plots showing tumour weights. (U) Slices show the cell proliferation of the xenografts, which were subject to cutting and detected by Ki67 antibody. Graphs show the level of Ki67, which was statistically calculated based on five slices. (V) Peritoneal metastasis model exhibited the migration ability of gastric carcinoma (GC) cells. Scatter plots showing the metastatic tumour number. In all figures, data are presented as mean ± standard error of the mean (SEM). The asterisks represented the statistical *p*‐value (*p < .05; **p < .01; ***p < .001; ****p < .0001; Student's test)

Figure S4. Corresponding to Figure 3. (A) Quantitative real‐time polymerase chain reaction (qRT‐PCR) was applied to detect the expression of cell cycle‐related genes expression in gastric carcinoma (GC) cells. β‐Actin served as the internal reference. (B) Cell cycle analysis was applied to detect the change of cell cycle when the lncSLCO1C1 was suppressed or overexpressed. (C) Red fluorescence shows the level of γH2AX in SGC7901 cells where lncSLCO1C1 was knocked down. DAPI indicates the cell nucleus. Bars show the intensity of red fluorescence, which was statistically calculated based on five slices. (D) Comet assay shows the damaged DNA in SGC7901 cells where lncSLCO1C1 expression was suppressed. Bars show the damaged DNA in the tail, which was statistically calculated based on three repeated biological experiments. (E) Yellow fluorescence shows the level of γH2AX in xenografts generated from SGC7901 cells where the expression of lncSLCO1C1 was decreased. Green fluorescent protein (GFP) indicates the expression of the sh‐lncSLCO1C1 vector in SGC7901 cells. DAPI indicates the cell nucleus. Bars show the intensity of yellow fluorescence, which was statistically calculated based on five slices. (F and G) Red fluorescence shows the level of γH2AX in SGC7901 and BGC823 cells where PDE3A was knocked down. DAPI indicates the cell nucleus. Bars show the intensity of red fluorescence, which was statistically calculated based on five slices. In all figures, data are presented as mean ± standard error of the mean (SEM). The asterisks represented the statistical *p*‐value (*p < .05; **p < .01; ***p < .001; ****p < .0001; Student's test)

Figure S5. Corresponding to Figure 4. (A and B) Proteins detected by mass spectrometer (MS) in the complex that was isolated from BGC823 and SGC7901 cells using RNA pull‐down experiments upon biotin‐labelled lncSLCO1C1, analysed by Panther (http://www.pantherdb.org/). (C) Co‐immunoprecipitation (Co‐IP) assay shows the interaction between structure‐specific recognition protein 1 (SSRP1) and H2A/H2B. (D) Western blotting shows SSRP1, H2A and H2B in the complex that was isolated from SGC7901 cells using RNA pull‐down experiments upon biotin‐labelled lncSLCO1C1. Sense indicates using the full‐length sequence of lncSLCO1C1. Antisense indicates using the inverse complementary sequence of lncSLCO1C1. (E) Graphs showing the enrichment of lncSLCO1C1 and lncRA1 by using anti‐H2B antibodies. The data are presented as mean ± standard error of the mean (SEM). The asterisks represented the statistical *p*‐value (*p < .05; **p < .01; ***p < .001; ****p < .0001; Student's test). (F) Graphs showing the enrichment of lncSLCO1C1 in the complex that was isolated from SGC7901 cells using anti‐SSRP1, anti‐H2A or anti‐H2B antibodies, respectively. The data are presented as mean ± SEM. The asterisks represented the statistical *p*‐value (*p < .05; **p < .01; ***p < .001; ****p < .0001; whilst one‐way analysis of variance (ANOVA) test). (G) The prediction of the binding region of lncSLCO1C1 with SSRP1, H2A and H2B was carried out using catRAPID (http://service.tartaglialab.com/page/catrapid_group). (H) Three‐dimensional models showing the interaction between specific binding region of lncSLCO1C1 and SSRP1, H2A or H2B was analysed by using NPDock website (http://genesilico.pl/NPDock)

Figure S6. Oxaliplatin treatment increases the expression of lncSLCO1C1, which elevates the expression of structure‐specific recognition protein 1 (SSRP1) mRNA and protein in gastric carcinoma (GC) cells. (A) Green fluorescence shows the expression of lncSLCO1C1 in BGC823 cells treated with different concentrations of oxaliplatin. Graphs show the intensity of green fluorescence that was statistically calculated based on five slices. (B) Quantitative real‐time polymerase chain reaction (qRT‐PCR) experiments show the expression of SLCO1C1 in BGC823 cells treated with increase concentrations of oxaliplatin. (C) Western blotting shows the expression of SSRP1 and GAPDH (as the internal reference) in BGC823 cells treated with different concentrations of oxaliplatin. (D) Western blotting shows the expression of SSRP1 and GAPDH (as the internal reference) in BGC823 cells that were treated with different concentrations of oxaliplatin and where lncSLCO1C1 was knocked down. (E–G) The expression of SSRP1 mRNA (E) and lncSLCO1C1 (G) in BGC823 cells where lncSLCO1C1 and SSRP1 (F) were knocked down, respectively. β‐Actin served as the internal reference. (H) The copy number of lncSLCO1C1, miR‐211‐5p and miR‐204‐5p in SGC7901 and BGC823 cells were detected using qRT‐PCR. (I) The expression of miR‐204‐5p and miR‐211‐5p were detected when oxaliplatin was added. (J and K) Red fluorescence shows the level of γH2AX in SGC7901 cells where miR‐204‐5p and miR‐211‐5p were added, without (J) or with (K) oxaliplatin treatment. DAPI indicates the cell nucleus. Bars show the intensity of red fluorescence, which was statistically calculated based on five slices. (L) The expression of potential target genes was detected by qRT‐PCR assay when lncSLCO1C1 was suppressed. In all figures, data are presented as mean ± standard error of the mean (SEM). The asterisks represented the statistical *p*‐value (*p < .05; **p < .01; ***p < .001; ****p < .0001; Student's test)

Figure S7 lncSLCO1C1/miR‐211‐5p/miR‐204‐5p regulates proliferation and metastasis of gastric carcinoma (GC) cells. (A and F) CCK‐8 assay was used to detect the proliferation ability of GC cells when miR‐204‐5p and miR‐211‐5p were added in SGC7901 (A) and BGC823 (F). (B and G) Colony assay was used to detect the proliferation ability of GC cells when miR‐204‐5p and miR‐211‐5p were added in SGC7901 (B) and BGC823 (G). (C and H) EDU assay was used to detect the proliferation ability of GC cells when miR‐204‐5p and miR‐211‐5p were added in SGC7901 (C) and BGC823 (H). (D and I) Transwell assay was used to detect the migration ability of GC cells when miR‐204‐5p and miR‐211‐5p were added in SGC7901 (D) and BGC823 (I). (E and J) The invasion ability of GC cells was determined by Matrigel Transwell assay, when miR‐204‐5p and miR‐211‐5p were added in SGC7901 (E) and BGC823 (J). (K and P) The proliferation ability was detected in SGC7901 (K) and BGC823 (P) by CCK‐8 assay, when lncSLCO1C1 suppression or lncSLCO1C1 suppression but miR‐204‐5p and miR‐211‐5p inhibitors were added. (L and Q) The proliferation ability was detected in SGC7901 (L) and BGC823 (Q) by Colony assay, when lncSLCO1C1 suppression or lncSLCO1C1 suppression but miR‐204‐5p and miR‐211‐5p inhibitors were added. (M and R) The proliferation ability was detected in SGC7901 (M) and BGC823 (R) by EDU assay, when lncSLCO1C1 suppression or lncSLCO1C1 suppression but miR‐204‐5p and miR‐211‐5p inhibitors were added. (N and S) The migration ability was detected in SGC7901 (N) and BGC823 (S) by Transwell assay, when lncSLCO1C1 suppression or lncSLCO1C1 suppression but miR‐204‐5p and miR‐211‐5p inhibitors were added. (O and T) The invasion ability was detected in SGC7901 (O) and BGC823 (T) by Matrigel Transwell assay, when lncSLCO1C1 suppression or lncSLCO1C1 suppression but miR‐204‐5p and miR‐211‐5p inhibitors were added. (U–W) The proliferation ability was detected in MKN28 by CCK‐8 assay, Colony assay and EDU assay, when lncSLCO1C1 overexpression or lncSLCO1C1 overexpression but miR‐204‐5p and miR‐211‐5p mimics were added. (X and Y) The metastasis of MKN28 cells was detected by Transwell assay (X) and Matrigel Transwell assay (Y), when lncSLCO1C1 overexpression or lncSLCO1C1 overexpression but miR‐204‐5p and miR‐211‐5p mimics were added. In all figures, data are presented as mean ± standard error of the mean (SEM). The asterisks represented the statistical *p*‐value (*p < .05; **p < .01; ***p < .001; ****p < .0001; Student's test)

Table S1. Clinical features of lncSLCO1C1 in Cohort 1

Table S2. Clinical features of lncSLCO1C1 in Cohort 2

Table S3. The detail information of lncSLCO1C1 in long non‐coding RNA (lncRNA) databases

Table S4. Protein coding capacity of lncSLCO1C1 in LNCipedia database

Table S5. Characteristic of lncSLCO1C1 in ORF Finder

Data S1. Sequence data of miRNA which was pulled down by lncSLCO1C1 and miRNA predicted binding with structure‐specific recognition protein 1 (SSRP1) 3′UTR.

Data S2. The primers for RACE assay and copy number assays.

Data S3. The specific primers of quantitative real‐time polymerase chain reaction (qRT‐PCR) assay.

Data S4. The sequence of shRNAs, probes, plasmids and antisense oligonucleotides (ASOs) applied in the study.

Data S5. The mRNA expression in BGC823 was detected by microarray after lncSLCO1C1 was suppressed.

Data S6. Mass spectrometer (MS) result of proteins which were pulled down by lncSLCO1C1 in SGC7901 and BGC823 cell.
