# Supplementary figures and images for: Role of lncSLCO1C1 in gastric cancer progression and resistance to oxaliplatin therapy
Source: Clin Transl Med. 2022 Apr 26;12(4):e691. doi: 10.1002/ctm2.691 (PMC9043116; doi:10.1002/ctm2.691)

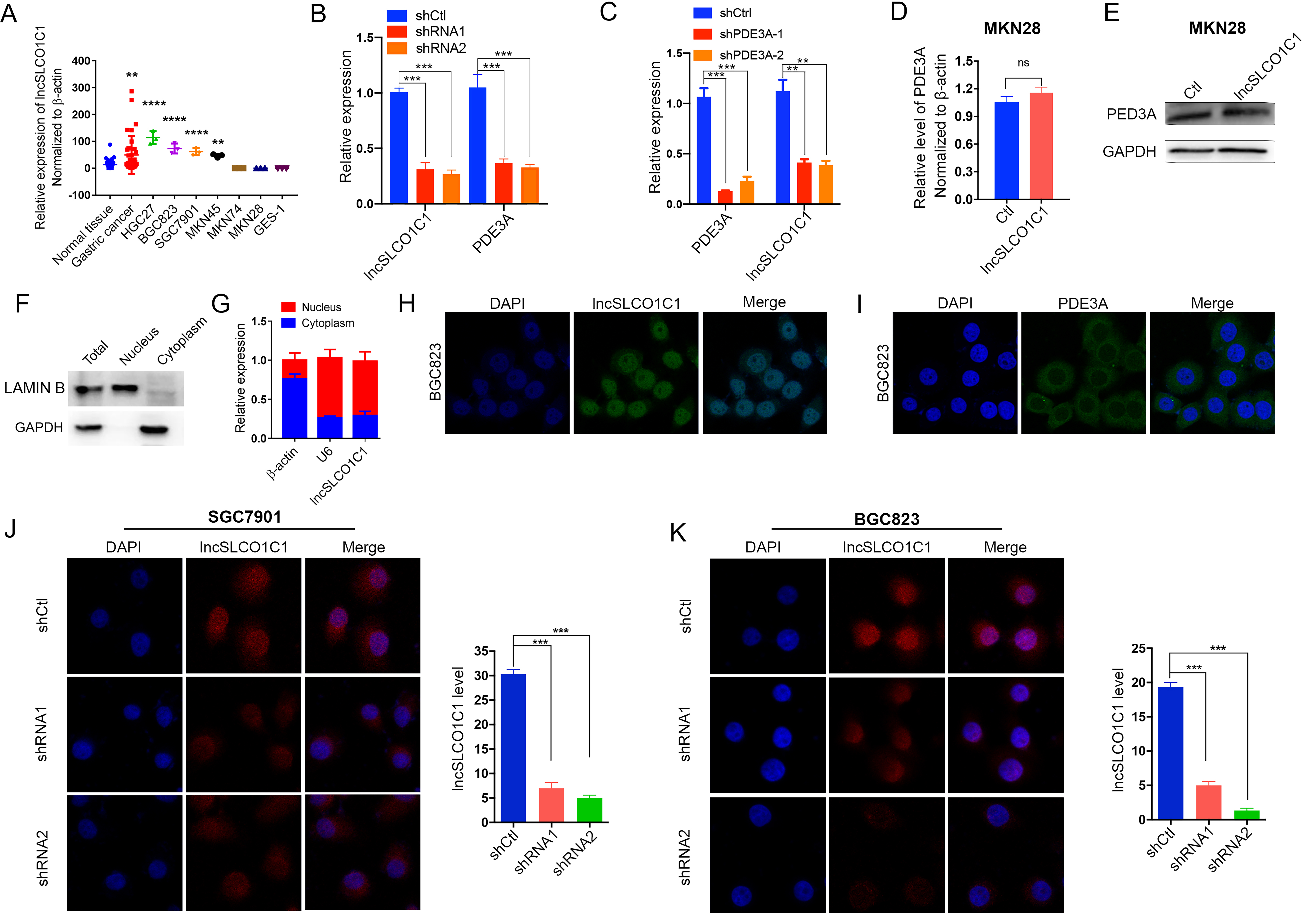

Supplement: Supplementary file 2 — Figure S2. Corresponding to Figure 2. (A) Scatter plots show the expression of lncSLCO1C1 in 49 pairs of gastric carcinoma (GC) and adjacent normal tissues and different GC cell lines. β‐Actin served as the internal reference. **p < .01, adjacent normal tissues were listed as control sample. The data are presented as mean ± standard error of the mean (SEM). The asterisks represented the statistical p‐value (*p < .05; **p < .01; ***p < .001; ****p < .0001; whilst one‐way analysis of variance (ANOVA) test). (B) Bars show the expression of lncSLCO1C1 and PDE3A mRNA in SGC7901 cells which were transfected with sh‐lncSLCO1C1 vectors. β‐Actin served as the internal reference. The data are presented as mean ± SEM. The asterisks represented the statistical p‐value (*p < .05; **p < .01; ***p < .001; ****p < .0001; whilst ANOVA test). (C) The expression of lncSLCO1C1 and PDE3A whilst PDE3A was suppressed. The data are presented as mean ± SEM. The asterisks represented the statistical p‐value (*p < .05; **p < .01; ***p < .001; ****p < .0001; whilst ANOVA test). (D and E) The mRNA and protein level of PDE3A in MKN28 cells which were transfected with lncSLCO1C1‐overexpressing vectors. The data are presented as mean ± SEM. The asterisks represented the statistical p‐value (*p < .05; **p < .01; ***p < .001; ****p < .0001; Student's test). (F) Nuclear plasma separation assay was performed in BGC823 cells, and Western blot assay was applied to measure the quality. (G) Graphs showing the distribution of β‐actin, U6 and lncSLCO1C1 in BGC823 cells. (H and I) The location of lncSLCO1C1 and PDE3A detected by fluorescence in situ hybridisation (FISH). (J and K) The expression and location of lncSLCO1C1 detected by FISH, when shRNAs are applied in SGC7901 and BGC823. Red colour shows the level and location of lncSLCO1C1. Blue colour indicates the cell nucleus stained by DAPI. The fluorescence intensity was analysed using Image J software. The data are presented as mean ± SEM. The asterisks [file CTM2-12-e691-s017.jpg]

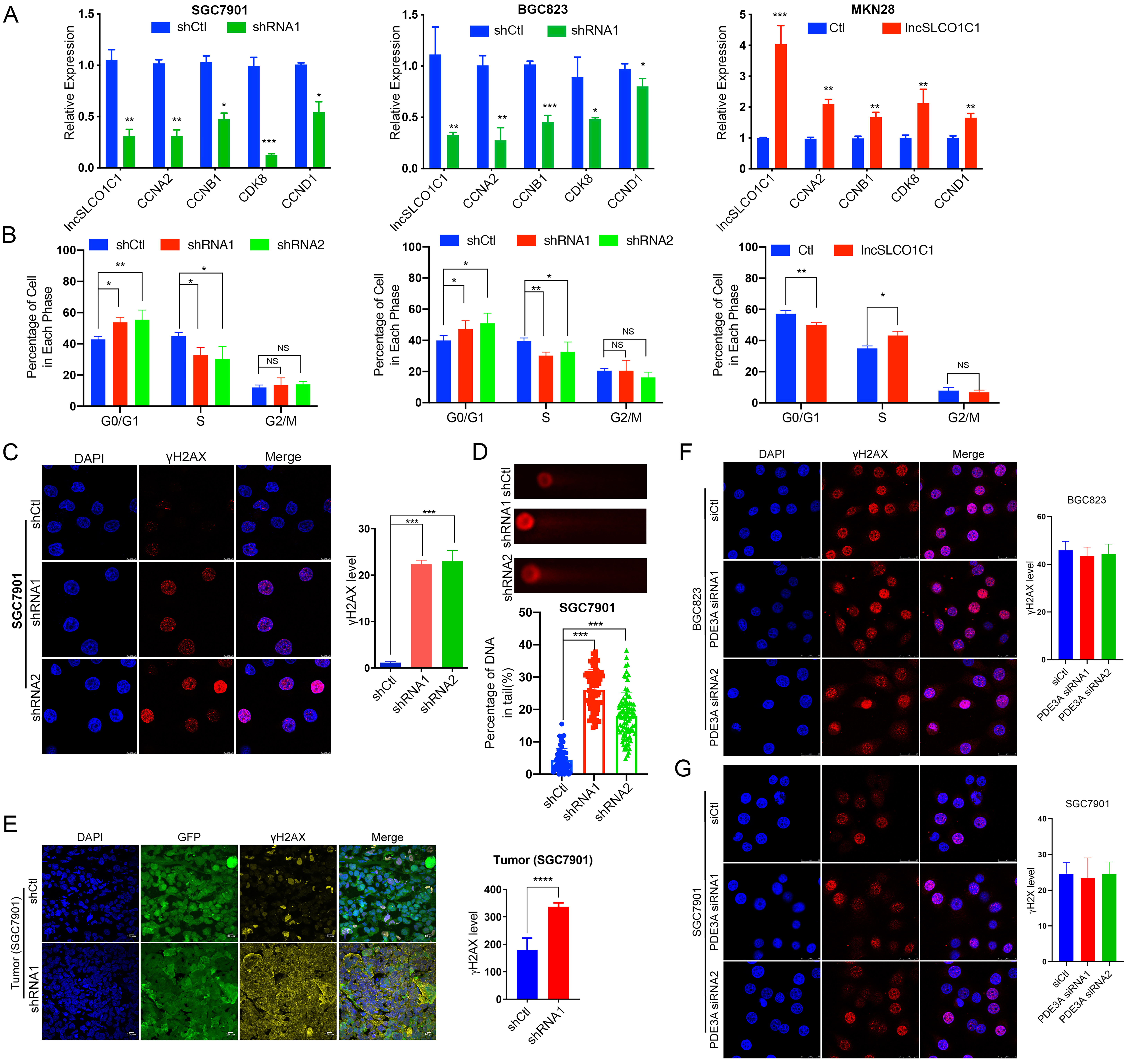

Supplement: Supplementary file 4 — Figure S4. Corresponding to Figure 3. (A) Quantitative real‐time polymerase chain reaction (qRT‐PCR) was applied to detect the expression of cell cycle‐related genes expression in gastric carcinoma (GC) cells. β‐Actin served as the internal reference. (B) Cell cycle analysis was applied to detect the change of cell cycle when the lncSLCO1C1 was suppressed or overexpressed. (C) Red fluorescence shows the level of γH2AX in SGC7901 cells where lncSLCO1C1 was knocked down. DAPI indicates the cell nucleus. Bars show the intensity of red fluorescence, which was statistically calculated based on five slices. (D) Comet assay shows the damaged DNA in SGC7901 cells where lncSLCO1C1 expression was suppressed. Bars show the damaged DNA in the tail, which was statistically calculated based on three repeated biological experiments. (E) Yellow fluorescence shows the level of γH2AX in xenografts generated from SGC7901 cells where the expression of lncSLCO1C1 was decreased. Green fluorescent protein (GFP) indicates the expression of the sh‐lncSLCO1C1 vector in SGC7901 cells. DAPI indicates the cell nucleus. Bars show the intensity of yellow fluorescence, which was statistically calculated based on five slices. (F and G) Red fluorescence shows the level of γH2AX in SGC7901 and BGC823 cells where PDE3A was knocked down. DAPI indicates the cell nucleus. Bars show the intensity of red fluorescence, which was statistically calculated based on five slices. In all figures, data are presented as mean ± standard error of the mean (SEM). The asterisks represented the statistical p‐value (*p < .05; **p < .01; ***p < .001; ****p < .0001; Student's test) [file CTM2-12-e691-s014.jpg]

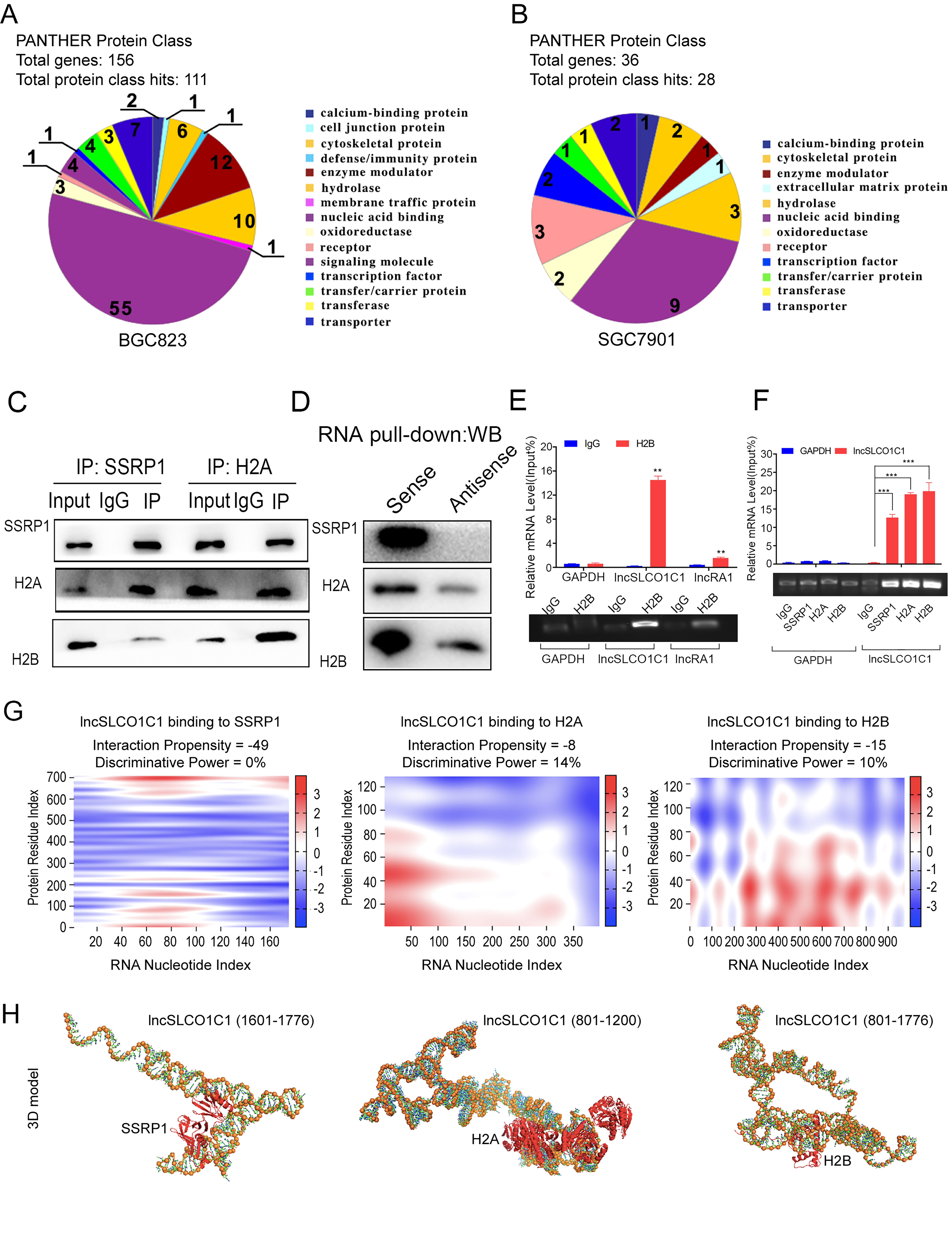

Supplement: Supplementary file 5 — Figure S5. Corresponding to Figure 4. (A and B) Proteins detected by mass spectrometer (MS) in the complex that was isolated from BGC823 and SGC7901 cells using RNA pull‐down experiments upon biotin‐labelled lncSLCO1C1, analysed by Panther (http://www.pantherdb.org/). (C) Co‐immunoprecipitation (Co‐IP) assay shows the interaction between structure‐specific recognition protein 1 (SSRP1) and H2A/H2B. (D) Western blotting shows SSRP1, H2A and H2B in the complex that was isolated from SGC7901 cells using RNA pull‐down experiments upon biotin‐labelled lncSLCO1C1. Sense indicates using the full‐length sequence of lncSLCO1C1. Antisense indicates using the inverse complementary sequence of lncSLCO1C1. (E) Graphs showing the enrichment of lncSLCO1C1 and lncRA1 by using anti‐H2B antibodies. The data are presented as mean ± standard error of the mean (SEM). The asterisks represented the statistical p‐value (*p < .05; **p < .01; ***p < .001; ****p < .0001; Student's test). (F) Graphs showing the enrichment of lncSLCO1C1 in the complex that was isolated from SGC7901 cells using anti‐SSRP1, anti‐H2A or anti‐H2B antibodies, respectively. The data are presented as mean ± SEM. The asterisks represented the statistical p‐value (*p < .05; **p < .01; ***p < .001; ****p < .0001; whilst one‐way analysis of variance (ANOVA) test). (G) The prediction of the binding region of lncSLCO1C1 with SSRP1, H2A and H2B was carried out using catRAPID (http://service.tartaglialab.com/page/catrapid_group). (H) Three‐dimensional models showing the interaction between specific binding region of lncSLCO1C1 and SSRP1, H2A or H2B was analysed by using NPDock website (http://genesilico.pl/NPDock) [file CTM2-12-e691-s006.jpg]

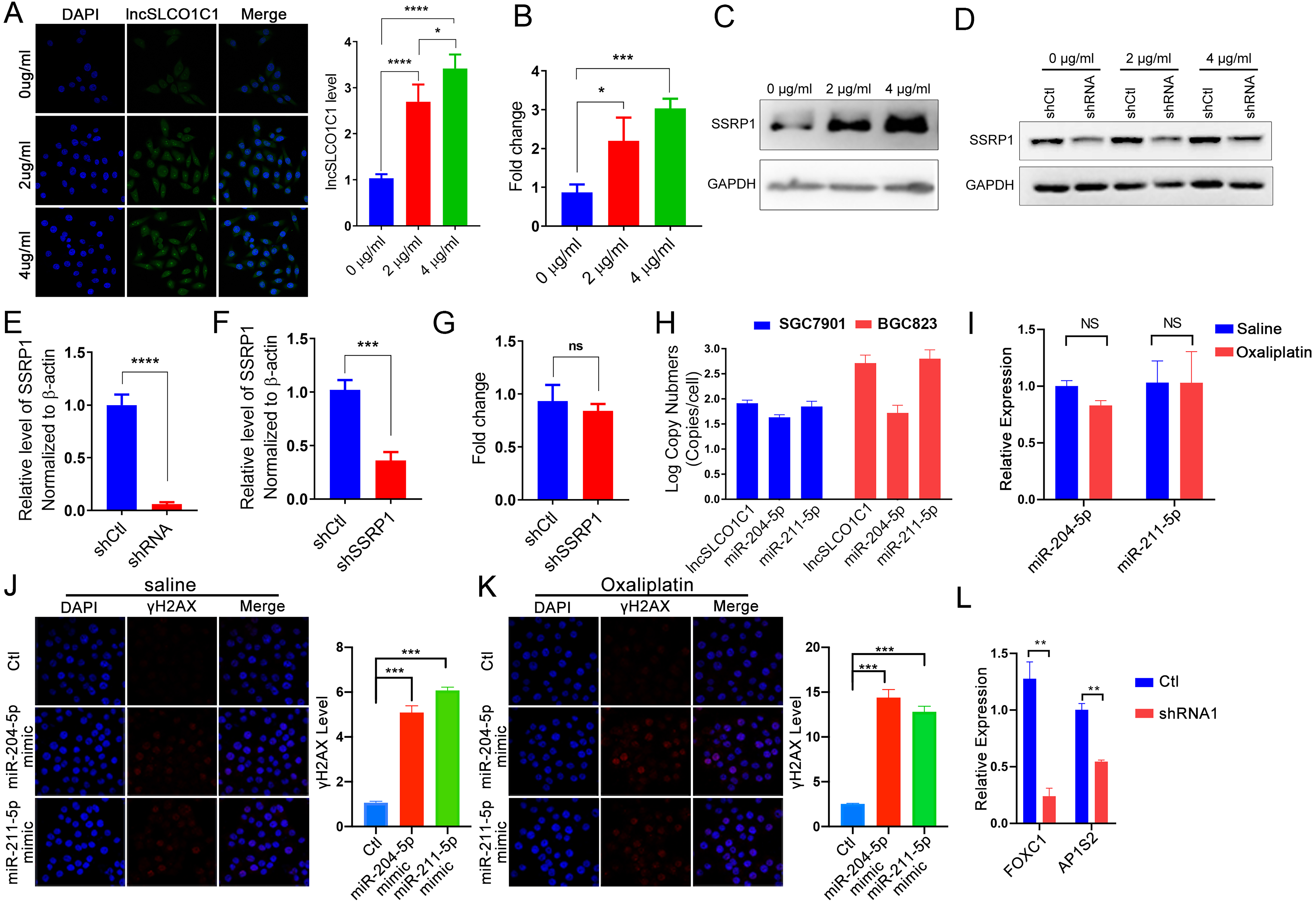

Supplement: Supplementary file 6 — Figure S6. Oxaliplatin treatment increases the expression of lncSLCO1C1, which elevates the expression of structure‐specific recognition protein 1 (SSRP1) mRNA and protein in gastric carcinoma (GC) cells. (A) Green fluorescence shows the expression of lncSLCO1C1 in BGC823 cells treated with different concentrations of oxaliplatin. Graphs show the intensity of green fluorescence that was statistically calculated based on five slices. (B) Quantitative real‐time polymerase chain reaction (qRT‐PCR) experiments show the expression of SLCO1C1 in BGC823 cells treated with increase concentrations of oxaliplatin. (C) Western blotting shows the expression of SSRP1 and GAPDH (as the internal reference) in BGC823 cells treated with different concentrations of oxaliplatin. (D) Western blotting shows the expression of SSRP1 and GAPDH (as the internal reference) in BGC823 cells that were treated with different concentrations of oxaliplatin and where lncSLCO1C1 was knocked down. (E–G) The expression of SSRP1 mRNA (E) and lncSLCO1C1 (G) in BGC823 cells where lncSLCO1C1 and SSRP1 (F) were knocked down, respectively. β‐Actin served as the internal reference. (H) The copy number of lncSLCO1C1, miR‐211‐5p and miR‐204‐5p in SGC7901 and BGC823 cells were detected using qRT‐PCR. (I) The expression of miR‐204‐5p and miR‐211‐5p were detected when oxaliplatin was added. (J and K) Red fluorescence shows the level of γH2AX in SGC7901 cells where miR‐204‐5p and miR‐211‐5p were added, without (J) or with (K) oxaliplatin treatment. DAPI indicates the cell nucleus. Bars show the intensity of red fluorescence, which was statistically calculated based on five slices. (L) The expression of potential target genes was detected by qRT‐PCR assay when lncSLCO1C1 was suppressed. In all figures, data are presented as mean ± standard error of the mean (SEM). The asterisks represented the statistical p‐value (*p < .05; **p < .01; ***p < .001; ****p < .0001; Student's test) [file CTM2-12-e691-s008.jpg]
