# Supplementary material for: Role of lncSLCO1C1 in gastric cancer progression and resistance to oxaliplatin therapy
Source: Clin Transl Med. 2022 Apr 26;12(4):e691. doi: 10.1002/ctm2.691 (PMC9043116; doi:10.1002/ctm2.691)
Supplement: Supplementary file 8 — Table S1. Clinical features of lncSLCO1C1 in Cohort 1 [file CTM2-12-e691-s016.docx]

Table S1 The analysis of lncSLCO1C1 based on the clinical data in cohort 1.

| **Clinical Pathology Data** | **Expression** | **P value** |
| --- | --- | --- |
| **Gender** |  |  |
| Male | 6.473 ± 2.358 | 0.4323 |
| female | 9.544 ± 3.17 |  |
| **Age** |  |  |
| ≧60 | 6.241 ± 2.839 | 0.6612 |
| <60 | 8.152 ± 2.496 |  |
| **Lymphatic metastasis** |  |  |
| Yes | 8.334 ± 2.239 | 0.4022 |
| No | 4.391 ± 2.31 |  |
| **Distant metastasis** |  |  |
| Yes | 9.408 ± 2.29 | 0.1032 |
| No | 1.251 ± 0.5628 |  |
| **Size** |  |  |
| ≧20cm^3^ | 11.57 ± 2.622 | **0.0414** |
| <20cm^3^ | 1.269 ± 0.4986 |  |
| **Differentiation** |  |  |
| Poorly Differentiation | 11.05 ± 2.426 | **0.0469** |
| Well Differentiation | 0.9145 ± 0.3111 |  |
| **Stage** |  |  |
| I/II | 5.511 ± 1.827 | 0.7854 |
| III/IV | 7.017 ± 5.208 |  |
