# Supplementary material for: Role of lncSLCO1C1 in gastric cancer progression and resistance to oxaliplatin therapy
Source: Clin Transl Med. 2022 Apr 26;12(4):e691. doi: 10.1002/ctm2.691 (PMC9043116; doi:10.1002/ctm2.691)
Supplement: Supplementary file 9 — Table S2. Clinical features of lncSLCO1C1 in Cohort 2 [file CTM2-12-e691-s003.docx]

Table S2 The analysis of lncSLCO1C1 based on the clinical data in cohort 2, n=90.

| **Clinical Pathology Data** | **Expression** | **P value** |
| --- | --- | --- |
| **Gender** |  |  |
| Male | 21.05 ± 0.5805 | 0.5407 |
| female | 21.73 ± 0.9395 |  |
| **Age** |  |  |
| ≧60 | 21.98 ± 0.6795 | 0.0812 |
| <60 | 20.25 ± 0.6796 |  |
| **Lymphatic metastasis** |  |  |
| Yes | 21.61 ± 0.6260 | 0.2044 |
| No | 20.20 ± 0.6252 |  |
| **Size** |  |  |
| ≧20cm^3^ | 21.80 ± 1.048 | **0.0404** |
| <20cm^3^ | 19.90 ± 0.3978 |  |
| **Differentiation** |  |  |
| Poorly Differentiation | 22.11 ± 0.6653 | **0.0209** |
| Well Differentiation | 19.46 ± 0.5574 |  |
| **Stage** |  |  |
| I/II | 21.29 ± 0.6421 | 0.9274 |
| III/IV | 21.2 ± 0.7139 |  |
