# Supplementary material for: Role of lncSLCO1C1 in gastric cancer progression and resistance to oxaliplatin therapy
Source: Clin Transl Med. 2022 Apr 26;12(4):e691. doi: 10.1002/ctm2.691 (PMC9043116; doi:10.1002/ctm2.691)
Supplement: Supplementary file 10 — Table S3. The detail information of lncSLCO1C1 in long non‐coding RNA (lncRNA) databases [file CTM2-12-e691-s009.docx]

Table S3 lncRNA SLCO1C1 in lncRNA database

| Database | Location(hg19) | Gene ID | Length | Strand |
| --- | --- | --- | --- | --- |
| LNCipedia | chr12:20835231-20837006 | lnc-SLCO1C1-2 | 1776 | + |
| Noncode | chr12:20835230-20837006 | NONHSAT027230 | 1776 | + |
