# Supplementary material for: Role of lncSLCO1C1 in gastric cancer progression and resistance to oxaliplatin therapy
Source: Clin Transl Med. 2022 Apr 26;12(4):e691. doi: 10.1002/ctm2.691 (PMC9043116; doi:10.1002/ctm2.691)
Supplement: Supplementary file 11 — Table S4. Protein coding capacity of lncSLCO1C1 in LNCipedia database [file CTM2-12-e691-s018.docx]

Table S4 The coding potential of lncRNA SLCO1C1 in LNCipedia database

| Metric | Raw result | Interpretaion |
| --- | --- | --- |
| PRIDE reprocessing 2.0 | 0 | noncoding |
| Lee translation initiation sites | 0 | noncoding |
| PhyloCSF score | -38.3481 | noncoding |
| CPAT coding probability | 0.32% | noncoding |
| Bazzini small ORFs | 0 | noncoding |
