# Supplementary material for: Role of lncSLCO1C1 in gastric cancer progression and resistance to oxaliplatin therapy
Source: Clin Transl Med. 2022 Apr 26;12(4):e691. doi: 10.1002/ctm2.691 (PMC9043116; doi:10.1002/ctm2.691)
Supplement: Supplementary file 12 — Table S5. Characteristic of lncSLCO1C1 in ORF Finder [file CTM2-12-e691-s005.docx]

Table S5 Characteristic of lncRNA SLCO1C1 in ORF finder

| Label | Strand | Frame | Start | Stop | Length(nt\|aa) | Protein Blast |
| --- | --- | --- | --- | --- | --- | --- |
| ORF1 | + | 1 | 550 | 705 | 156\|51 | No putative conserved domains have been detected |
| ORF2 | + | 2 | 53 | 130 | 78\|25 | No putative conserved domains have been detected |
| ORF3 | + | 2 | 1244 | 1360 | 117\|38 | No significant similarity found |
| ORF4 | + | 3 | 357 | 539 | 183\|60 | No significant similarity found |
| ORF5 | + | 3 | 969 | 1085 | 117\|38 | No putative conserved domains have been detected |
| ORF6 | + | 3 | 1440 | 1601 | 162\|53 | No significant similarity found |
| ORF7 | - | 1 | 1356 | 1189 | 168\|55 | No significant similarity found |
| ORF8 | - | 1 | 84 | >1 | 84\|27 | No putative conserved domains have been detected |
| ORF9 | - | 3 | 526 | 446 | 81\|26 | No putative conserved domains have been deteceted |
